# Orthogonal transcriptional modulation and gene editing using multiple CRISPR-Cas systems

**DOI:** 10.1016/j.ymthe.2024.11.024

**Published:** 2024-11-19

**Authors:** Amalie Dyrelund Broksø, Louise Bendixen, Simon Fammé, Kasper Mikkelsen, Trine Ilsø Jensen, Rasmus O. Bak

**Affiliations:** 1 Department of Biomedicine, Aarhus University, Aarhus C, Denmark

**Keywords:** CRISPRa, CRISPRi, transcriptional, activation, repression, orthogonal, Cas, orthologs, trimodal, CRISPR

## Abstract

CRISPR-Cas-based transcriptional activation (CRISPRa) and interference (CRISPRi) enable transient programmable gene regulation by recruitment or fusion of transcriptional regulators to nuclease-deficient Cas (dCas). Here, we expand on the emerging area of transcriptional engineering and RNA delivery by benchmarking combinations of RNA-delivered dCas and transcriptional modulators. We utilize dCas9 from *Staphylococcus aureus* and *Streptococcus pyogenes* for orthogonal transcriptional modulation to upregulate one set of genes while downregulating another. We also establish trimodal genetic engineering by combining orthogonal transcriptional regulation with gene knockout by Cas12a (*Acidaminococcus*; AsCas12a) ribonucleoprotein delivery. To simplify trimodal engineering, we explore optimal parameters for implementing truncated single guide RNAs (sgRNAs) to make use of SpCas9 for knockout and CRISPRa. We find the Cas9 protein/sgRNA ratio to be crucial for avoiding sgRNA cross-complexation and for balancing knockout and activation efficiencies. We demonstrate high efficiencies of trimodal genetic engineering in primary human T cells while preserving basic T cell health and functionality. This study highlights the versatility and potential of complex genetic engineering using multiple CRISPR-Cas systems in a simple one-step process yielding transient transcriptome modulation and permanent DNA changes. We believe such elaborate engineering can be implemented in regenerative medicine and therapies to facilitate more sophisticated treatments.

## Graphical abstract

**Figure:**
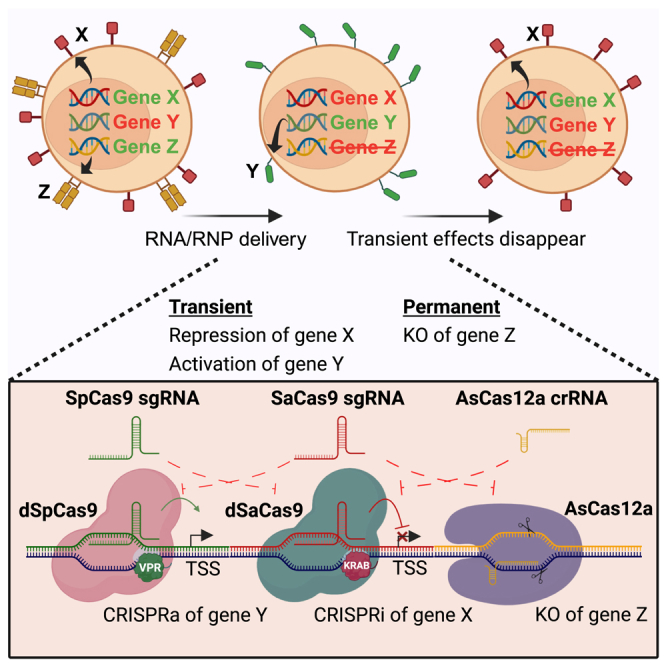

## Teaser

Bak and colleagues showcase complex genetic engineering of cells with simultaneous transient upregulation of one gene, transient downregulation of a second gene, and permanent knockout of a third gene. Such sophisticated engineering can be implemented within areas of gene and cell therapy.

## Introduction

**Figure 1:**
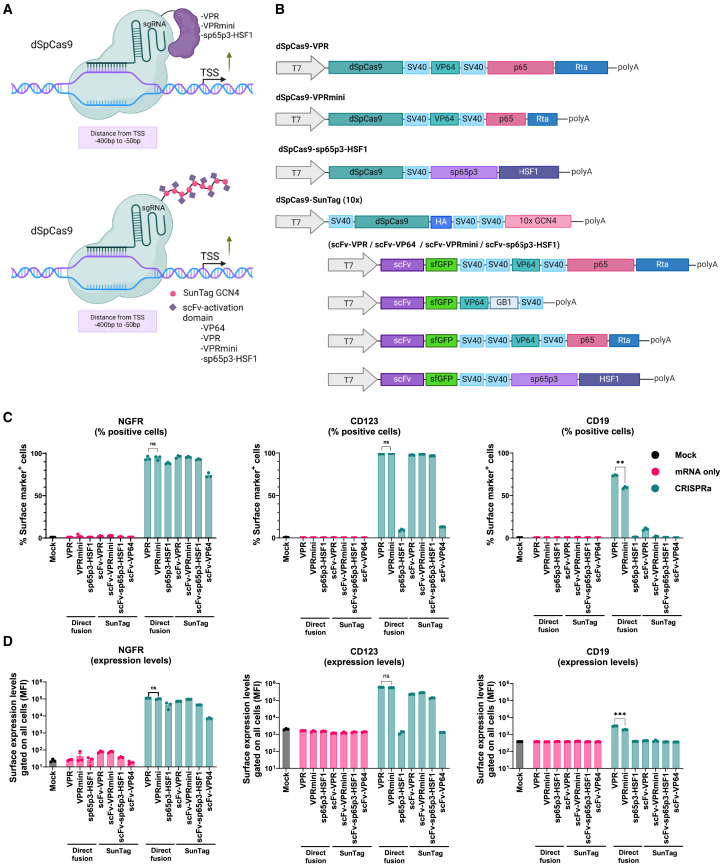
Screening of all-RNA CRISPRa effectors in Jurkat cells (A) Schematic representation of CRISPRa systems composed of dSpCas9 with either direct fusion to effector domains (top) or to the SunTag system (bottom) to activate transcription. Position of the effector domain on dCas9 is illustrative and can be at either terminus of dCas9. The ideal targeting window relative to the transcriptional start site (TSS) is shown below the DNA. (B) Schematic representation of the IVT templates for each CRISPRa system. SV40 is a nuclear localization signal (NLS), GB1 is a small solubility tag, 10x GCN4 is the antigen scaffold for scFv-effector recruitment, and sfGFP is superfolder GFP, which enhances scFv-GCN4 stability. Element sizes are not drawn to scale. (C and D) CRISPRa in Jurkat cells of *NGFR* (left), *CD123* (middle), and *CD19* (right) with (C) percentage of positive cells or (D) median fluorescence intensity (MFI) analyzed by flow cytometry 24 h post electroporation with IVT mRNA for the selected CRISPRa system and chemically modified sgRNAs (four sgRNAs for each target). “mRNA only” samples (pink) are negative controls where sgRNAs were omitted. Data are presented as mean ± SEM, (*n* = 3). ns, not significant; ∗∗∗∗*p* ≤ 0.0001. *p* values were calculated using a one-way ANOVA with Tukey’s multiple comparisons test.

**Figure 2:**
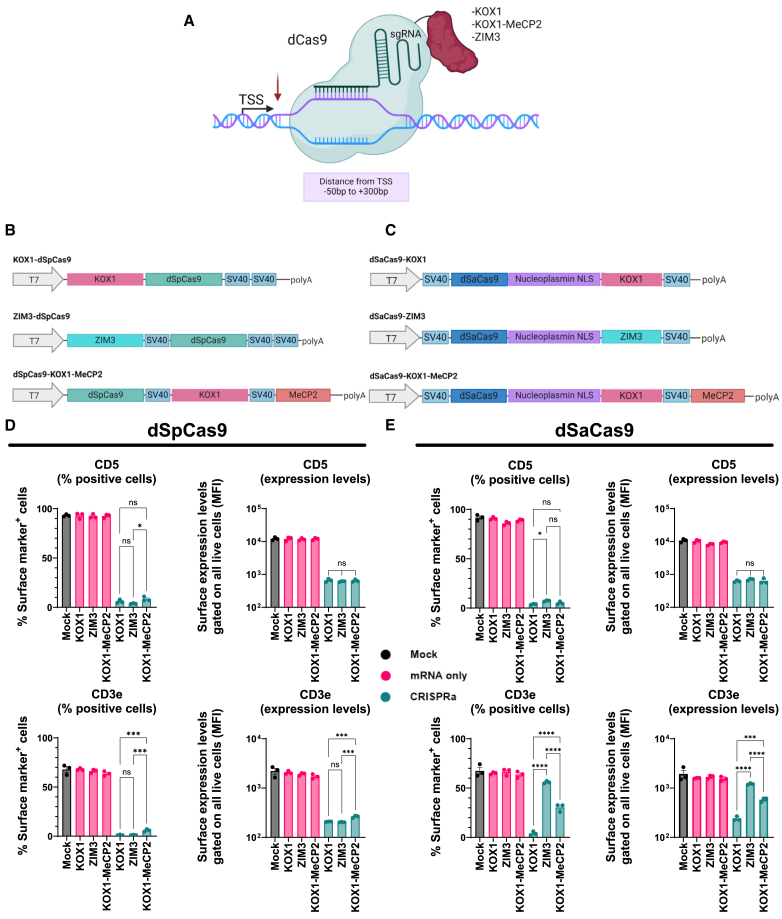
Comparison of CRISPRi effectors using all-RNA delivery in Jurkat cells (A) Schematic representation of the CRISPRi systems composed of dCas9 fused directly to effector domains. Position of the effector domain on dCas9 is illustrative and can be at either terminus of dCas9. The ideal targeting window relative to the transcriptional start site (TSS) is shown below the DNA. (B and C) Schematic representation of the IVT constructs for each dSpCas9 (B) and dSaCas9 (C) CRISPRi system. Element sizes are not drawn to scale. (D) dSpCas9 CRISPRi of *CD5* (top) and *CD3e* (bottom) with percentage of positive cells (left) and median fluorescence intensity (MFI) (right). (E) dSaCas9 CRISPRi of *CD5* (top) and *CD3e* (bottom) with percentage of positive cells (left) and median fluorescence intensity (MFI) (right). For data in (C) and (E), cells were analyzed by flow cytometry for surface protein expression 72 h post electroporation with IVT mRNA for the selected CRISPRi system and chemically modified sgRNAs for *CD5* (three for SaCas9 and four for SpCas9) and *CD3e* (three for both Sp- and SaCas9). “mRNA only” samples (pink) are negative controls where sgRNAs were omitted. Data are presented as mean ± SEM (*n* = 3). ns, not significant (*p* > 0.05); ∗*p* ≤ 0.05, ∗∗*p* ≤ 0.01, ∗∗∗*p* ≤ 0.001, ∗∗∗∗*p* ≤ 0.0001. *p* values were calculated using a one-way ANOVA with Tukey’s multiple comparisons test.

Clustered regularly interspaced short palindromic repeats (CRISPR)-Cas-mediated activation (CRISPRa) and interference (CRISPRi) are modified, nuclease-deficient molecular tools that enable precise and transient transcriptional modulation.^1^^,^^2^^,^^3^ CRISPRa and CRISPRi have been accomplished using various nuclease-deficient Cas (dCas) variants, most notably dCas9 derived from *Streptococcus pyogenes* (dSpCas9) and *Staphylococcus aureus* (dSaCas9).^4^ Various strategies have been employed for inducing or repressing transcription from the targeted gene (reviewed in Bendixen et al.^5^). In short, these strategies rely on either the direct fusion of transcriptional modulators to dCas proteins or recruitment of transcriptional modulators by different mechanisms (Figures 1A and 2A). For CRISPRa, a direct fusion to the tripartite activation domain VPR (VP64-p65-Rta) has proven very efficient in promoting potent upregulation, as has the combination of p65 and HSF1 when fused to dCas12a.^6^^,^^7^ For CRISPRi, the highly utilized KOX1 Krüppel-associated box (KRAB) domain or the ZIM3 KRAB domain interacts with KAP1 and lead to recruitment of histone methyltransferases and deacetylases, facilitating strong transcriptional repression.^8^^,^^9^ While KRAB is also utilized in combination with DNA methyltransferase domains such as DNMT3A and DNMT3L for epigenome editing that installs heritable repressive epigenetic marks, KRAB alone is mostly found to yield reversible gene repression.^10^ However, some divergence in the literature suggests some context-dependent deviations from this paradigm.^9^^,^^11^^,^^12^

dCas-effector proteins together with CRISPR-Cas guide RNAs (gRNAs) create simple two-component transcriptional modulation platforms where one or several gRNAs are designed to direct the dCas9 protein:RNA complex to the region of the transcriptional start site (TSS) of a target gene to modulate transcription. Three-component systems have also been developed for CRISPRa, namely the SunTag and synergistic activation mediator (SAM) systems.^13^^,^^14^ The SunTag system fuses dCas to an array of antigens, which are recognized by a cognate single-chain variable fragment (scFv). Fusing transcriptional activators, e.g., VP64, to the scFv facilitates recruitment of multiple VP64 copies to dCas. The SunTag system has also been reported with scFv-KOX1 fusions for CRISPRi.^15^ The SAM system utilizes modified SAM gRNAs in which hairpins in the scaffold region of the gRNA are expanded with aptamers that are recognized by the MS2 protein.^14^ Hence, fusing transcriptional activators, for example p65-HSP1, to MS2 leads to recruitment of these activators to the gRNA.

The delivery method used for these transcriptional modulators is crucial for efficiency and duration of the transcriptional impact, as these systems can be transient or permanent depending on whether the genetic elements encoding the effectors are chromosomally integrated or not, for example using lentiviral vectors.^16^^,^^17^ Alternatively, in non-dividing cells, episomal viral genomes from, for example, adeno-associated viral (AAV) vectors can persist and provide a durable effect.^18^ Plasmid delivery, a common transient method, needs time for both transcription and translation to occur and comes with the risk of random genomic integration, possibly leading to genotoxicity as well as undesired persistent expression of the dCas9-effector.^19^^,^^20^ We have previously shown that mRNA delivery by electroporation of the dCas-effector as *in vitro* transcribed (IVT) mRNA in conjunction with synthetic and chemically end-modified single guide RNAs (sgRNAs) allows for fast-acting, transient transcriptional modulation with no risk of DNA integration and little to no cellular toxicity in primary cells.^21^^,^^22^ Transfection of IVT mRNA by electroporation can target close to 100% of cells with very homogeneous gene-expression levels, in contrast to plasmid delivery, which suffers from suboptimal transfection rates and cytotoxicity in many cell types as well as heterogeneous expression levels.^22^

While many different CRISPR-Cas systems and transcriptional effectors have been reported to date, and some have been compared, such efforts have focused on plasmid delivery, and very little work has been done with all-RNA platforms. Here, we produce and characterize new combinations of Cas variants and transcriptional effectors for RNA delivery with the aim of producing compatible and optimized platforms for transient and simultaneous CRISPRa and CRISPRi, also known as orthogonal gene regulation. We also investigate parameters for the implementation of simultaneous gene editing for knockout, thereby paving the way for complex genetic and transcriptional engineering.

## Results

### Comparison of CRISPRa effectors using RNA delivery

The VPR fusion and SunTag systems (scFv-VP64, scFv-p65-HSF1) have previously been described and proven to be more effective than the original VP64 fusion, but to our knowledge only the VP64 and VPR fusions have been described for IVT mRNA delivery.^6^^,^^13^^,^^22^^,^^23^^,^^24^ All-RNA delivery of the SAM system is challenging, since SAM sgRNAs are at a length where chemical RNA synthesis is inconsistent. We have previously tested an SAM all-RNA system based on two-part gRNAs (crRNA + tracrRNA), but this was found to be inferior to the VPR fusion strategy.^22^ We therefore focused on the direct fusion and SunTag strategies to explore seven different CRISPRa constructs for RNA delivery (Figures 1A and 1B). In addition to dSpCas9-VPR, two of the constructs were direct fusions to VPRmini or sp65p3-HSF1, respectively, which have previously been reported for CRISPRa.^7^^,^^25^ A dSpCas9-SunTag construct was generated for use with four different scFv fusions: scFv-VP64, scFv-VPR, scFv-VPRmini, and scFV-sp65p3-HSF1. VPRmini is a minimal version of VPR, originally developed as a dSaCas9 fusion to reduce the size of the CRISPRa system.^25^ While VP64 and p65-HSF1 scFv fusions have previously been reported for the SunTag system, neither VPR nor VPRmini have been used in this context.^13^^,^^23^ The SunTag system has, to our knowledge, never been used for RNA delivery. As protein linkers and positions of nuclear localization signals (NLSs) can potentially affect both dCas9 DNA binding and transcriptional effector function, we maintained reported protein configurations and NLS numbers from the literature when available. For the three previously unreported scFv fusions, we maintained the C-terminal fusion as reported for VP64.^13^

mRNA encoding the seven different CRISPRa effectors was generated and tested by electroporation of the T lymphoblastic Jurkat cell line, with combined delivery of previously reported functional sgRNAs for targeting *NGFR*, *CD123*, or *CD19*, genes encoding surface proteins easily detectable by flow cytometry.^22^ For each gene, four sgRNAs were co-delivered, since additive activation has been observed with the use of multiple sgRNAs.^22^^,^^26^ Changes in target expression levels were determined by flow cytometry 24 h post electroporation (Figures 1C, 1D, and S1A). While all effector mRNAs were functional, as seen by the ability to activate *NGFR* transcription in at least 72% of cells across all CRISPRa constructs, large variation was observed across constructs and target genes. *NGFR* was easily activated, with four effectors yielding upregulation in nearly all cells with comparable NGFR expression levels. *CD19* was more difficult to activate, with the best effector (VPR) activating *CD19* in around 70% of cells and four effectors (sp65p3-HSF1, scFv-VPRmini, scFv-sp65p3-HSF1, and scFv-VP64) showing no upregulation at all. While dSpCas9-VPR and dSpCas9-VPRmini gave rise to complete and equal activation of *NGFR* and *CD123*, dSpCas9-VPR showed superior activation of *CD19*. The dSpCas9-sp65p3-HSF1 fusion achieved *NGFR* activation in ∼90% of cells, albeit yielding lower NGFR expression levels than dSpCas9-VPR and dSpCas9-VPRmini. However, dSpCas9-sp65p3-HSF1 achieved little to no *CD123* and *CD19* upregulation. For the SunTag systems, the scFv-VPR and scFv-VPRmini achieved levels of transcriptional activation of *NGFR* and *CD123* that were comparable to the two effectors in the fusion context, but little to no upregulation was observed for *CD19*. scFv-sp65p3-HSP1 and scFv-VP64 generally performed worse than scFv-VPR and scFv-VPRmini. Overall, the dSpCas9-VPR construct performed best across all three target genes and was therefore used for further CRISPRa experiments.

### Comparison of CRISPRi effectors using RNA delivery

For CRISPRi, KOX1 KRAB constitutes the most widely used KRAB domain and is often merely referred to as KRAB in previous literature. Here, we also include the ZIM3 KRAB domain so to clearly distinguish between the two, they are referred to as KOX1 and ZIM3. KOX1 has also been employed as a fusion protein with methyl CpG binding protein 2 (MeCP2).^27^ In a prior comparison using plasmid delivery, ZIM3 was reported to induce more potent repression than KOX1 and KOX1-MeCp2 when fused to dSpCas9.^9^ Only KOX1-dSpCas9 has been reported for RNA delivery.^22^ Hence, we tested these three CRISPRi effectors fused directly to dSpCas9 as all-RNA delivery by electroporation into Jurkat cells (Figures 2A and 2B). Here, we tested CRISPRi of the surface-expressed genes *CD5* and *CD3e* with assessment of target gene expression by flow cytometry 72 h post electroporation, as we have previously observed this to be the time of maximum repression in a similar hematopoietic cell line, K562.^22^ All three dSpCas9 constructs achieved very efficient repression of both target genes, with all three constructs leaving <10% of cells positive for CD5 or CD3e (Figures 2D and S1B). No statistically significant difference was observed between KOX1 and ZIM3, but KOX1-MeCP2 was slightly inferior to both individual repressors for *CD3e*. To expand on the comparison, we extended the analysis to CRISPRi of the *CD2* gene. Two separate CD2 populations in non-manipulated cells made it infeasible to track the CRISPRi effect on a single-cell level, but overall reduction in *CD2* expression levels as determined by median fluorescence intensity (MFI) showed efficient gene repression, again with comparable efficiencies across the three effectors (Figures S2A, S2B, and S2E).

To perform orthogonal CRISPR-Cas transcriptional modulation for simultaneous target-specific up- and downregulation of distinct genes, two different Cas orthologs are required to avoid cross-complexing of sgRNAs intended for CRISPRa and sgRNAs intended for CRISPRi. We therefore constructed dSaCas9 fusion variants of the three previous CRISPRi effectors, produced mRNA, and tested these for CRISPRi of *CD3e* and *CD5* in Jurkat cells analogously to what was done with dSpCas9 (Figures 2C–2E and S1C). As for dSpCas9, *CD5* was efficiently repressed with no discernible difference across the three effector fusions. For *CD3e*, all three dSaCas9-effector fusions were functional, but KOX1 led to a much more potent repression followed by KOX1-MeCP2. Further experiments targeting *CD2* showed the same hierarchy among the effectors, with KOX1 outperforming the other two (Figures S2C–S2E). As the dSaCas9-KOX1 construct showed the strongest gene repression for all target genes, this was chosen for further use in orthogonal transcriptional modulation.

### Multiplexed and orthogonal transcriptional modulation

**Figure 3:**
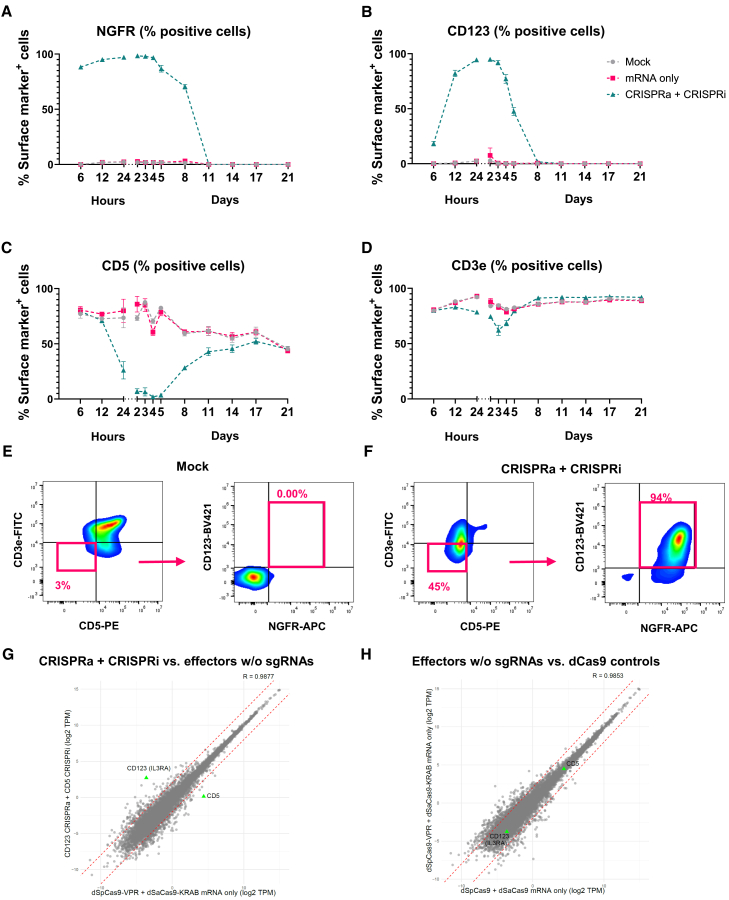
Orthologous, multiplexed, and transient CRISPRa and CRISPRi in Jurkat cells, and evaluation of off-target effects by RNA sequencing (A–D) Jurkat cells were electroporated with IVT mRNA for the CRISPRa dSpCas9-VPR system and CRISPRi dSaCas9-KOX1 system and chemically modified sgRNAs for all target genes (four for each of the CRISPRa targets and three for each of the CRISPRi targets). The percentage of cells positive for the four genes (A) *NGFR*, (B) *CD123*, (C) *CD5*, and (D) *CD3e* were assessed over a 21-day period at each of the indicated time points post electroporation using flow cytometry (*n* = 3). (E and F) Flow-cytometry plots from day 3 for the experiment described in (A)–(D) after either (E) mock electroporation or (F) electroporation with reagents for orthologous CRISPRa and CRISPRi. The red gates indicate the CD3e^−^ CD5^−^ and CD123^+^ NGFR^+^ populations (percentage of parent gate). The flow-cytometry plots are presented from the most efficient replicate. (G and H) Jurkat cells were electroporated with reagents for simultaneous *CD123* CRISPRa and *CD5* CRISPRi, and RNA was purified for RNA-seq on day 3 post electroporation. Cells receiving only dSpCas9-VPR + dSaCas9-KOX1 (mRNA only) were included as well as cells receiving dSpCas9 and dSaCas9 mRNAs without effectors. (G) Gene-expression levels (log_2_ transcripts per million [TPM]) in cells electroporated with reagents for *CD123* CRISPRa and *CD5* CRISPRi (y axis) vs. expression in cells electroporated with dSpCas9-VPR + dSaCas9-KOX1 mRNA only (x axis). (H) Gene-expression levels (log_2_TPM) in cells electroporated with dSpCas9-VPR mRNA + dSaCas9-KOX1 mRNA vs. cells electroporated with dSpCas9 mRNA + dSaCas9 mRNA without effectors. *R* indicates Pearson’s correlation coefficient calculated for log-transformed values on all genes except *CD123* and *CD5*. Genes with 0 TPM in either of the replicates were excluded before log transformation. Each data point represents the average TPM of biological triplicates (*n* = 3). Data in (A)–(D) are presented as mean ± SEM (*n* = 3).

Based on the previous experiments, the dSpCas9-VPR and dSaCas9-KOX1 effector fusions proved to induce the most potent transcriptional modulation when delivered as IVT mRNA along with synthetic sgRNAs (Figures 1, 2, S1, and S2). This allowed us to pursue multiplexed and orthogonal transcriptional modulation, dedicating the two Cas orthologs to CRISPRa and CRISPRi, respectively. *NGFR* and *CD123* were chosen for upregulation, while *CD3e* and *CD5* were selected for repression. Jurkat cells were electroporated with dSpCas9-VPR and dSaCas9-KOX1 mRNA and 3–4 sgRNAs per target. The efficiency and kinetics of transcriptional modulation were determined by flow cytometry at regular intervals between 6 h and 21 days post electroporation (Figures 3A–3D). Nearly all treated cells showed upregulation of *NGFR*. This upregulation was evident at 6 h post electroporation with peak activation between days 1–3 showing up to 99% NGFR^+^ cells. At 8 days post electroporation >80% of cells were still NGFR^+^, but this had declined to baseline at 11 days post electroporation. *CD123* activation showed a slower onset and shorter duration, with only ∼20% of cells showing *CD123* expression at 6 h post electroporation. This peaked at days 1–3, reaching up to 98% CD123^+^ cells, before declining to baseline at 8 days post electroporation. Downregulation of *CD3e* and *CD5* could be detected 24 h post electroporation with near full *CD5* repression lasting from day 2 to day 5 before gradually returning to normal expression levels at day 21. *CD3e* repression was not as effective in the multiplexed setting, with a reduction from about 75% CD3e^+^ cells to 50% at around 3 days post electroporation. This returned to baseline on day 5. Analysis of the CD3e^−^ CD5^−^ population on day 3 showed that most of these cells were also CD123^+^ NGFR^+^, confirming that the imposed quadruplex transcriptional engineering occurred in the same cells (Figures 3E and 3F). Overall, 85% of cells on day 3 displayed the desired gene-expression status of three of the four target genes (NGFR^+^ CD123^+^ CD5^−^) compared to 0.01% of mock-treated cells. For all four target genes, ∼36% of cells displayed the desired gene-expression status (NGFR^+^ CD123^+^ CD5^−^ CD3e^−^) compared to 0.01% for mock cells. This shows that multiplexed orthogonal CRISPRa and CRISPRi by mRNA delivery is feasible and could prove to be useful for transient transcriptional modulation of multiple target genes.

### Gene-expression profiling reveals targeted transcriptional modulation without detectable off-target effects

To assess the specificity of CRISPRa and CRISPRi, we performed orthogonal transcription regulation, upregulating *CD123* and downregulating *CD5* in Jurkat cells followed by RNA sequencing (RNA-seq) at day 3 post electroporation. This was the common time point when both genes were optimally modulated as shown in the previous experiment (Figures 3B and 3C). The scatterplot comparing transcripts for cells with *CD123* CRISPRa and *CD5* CRISPRi to cells only receiving effector mRNAs without sgRNAs (dSpCas9-VPR + dSaCas9-KRAB mRNA only) showed 75-fold upregulation of *CD123* and 19-fold downregulation of *CD5* (Figure 3G). The Pearson correlation coefficient in gene expression of *R* = 0.99 confirmed that global transcription was not widely affected by the orthogonal CRISPR system. Analysis of predicted off-target sites for the *CD123* and *CD5* sgRNAs using the CRISPOR and COSMID off-target prediction tools identified a combined total of 22 and 724 potential off-targets for the two tools, respectively (Table S5). sgRNAs for CRISPRa and CRISPRi should optimally be located within a window of 700 bp around the TSS of the target gene and rarely have activity beyond a window of 1,500 bp from the TSS.^16^ We therefore analyzed whether any of the predicted off-targets were situated within a 10,000-kb window of the TSS of any of the identified differentially expressed genes (DEGs), but the nearest off-target site was placed >22 kb from one. Interestingly, one of the DEGs was a long non-coding RNA, *LOC101928032*, transcribed from the same locus as *CD123* but in the opposite direction (Figure S3A). While their TSSs are 41.7 kb apart and the *CD123* sgRNAs should therefore not upregulate *LOC101928032*, we hypothesize that *CD123* activation could modify local chromatin structures that induce transcription of *LOC101928032*. However, the genes immediately neighboring *CD123* were not upregulated (Figure S3B). Together, these findings suggest high specificity of both the CRISPRa and CRISPRi systems for the sgRNAs and indicate that the DEGs are likely the result of the biological impact of regulating *CD123* and *CD5* expression.

To evaluate whether overexpression of the two strong transcriptional effectors, VPR and KOX1, would have an sgRNA-independent impact on gene expression, we further compared gene expression in Jurkat cells receiving effector mRNAs without sgRNAs (dSpCas9-VPR + dSaCas9-KOX1 mRNA only) to cells receiving dCas9 mRNAs without effectors or sgRNAs (dSpCas9 + dSaCas9 mRNA only). Gene-expression comparison showed no pronounced effect on global gene expression, with a Pearson correlation coefficient of *R* = 0.99 (Figure 3H).

### Trimodal genetic engineering with simultaneous gene activation, repression, and knockout

**Figure 4:**
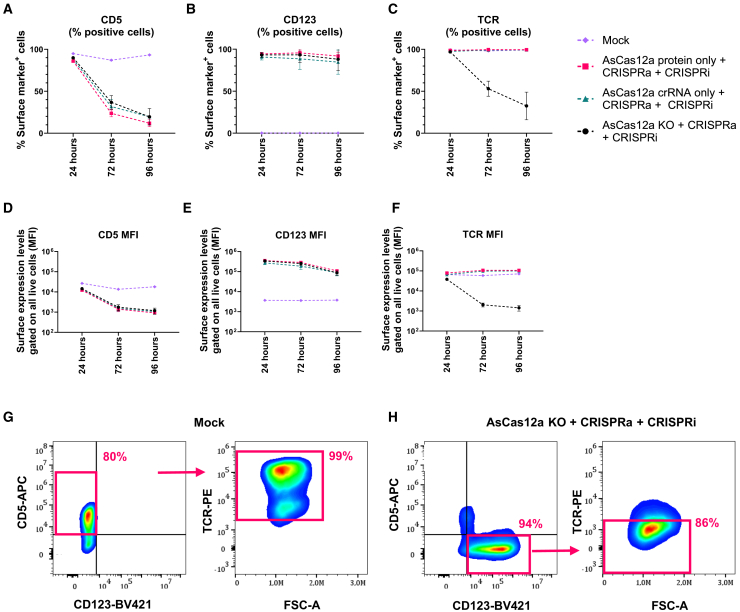
Simultaneous CRISPRa, CRISPRi, and knockout using three Cas orthologs (A–C) Jurkat cells were electroporated with reagents for AsCas12a-mediated *TRAC* knockout (AsCas12a KO), *CD123* CRISPRa (dSpCas9-VPR + *CD123* sgRNAs), and *CD5* CRISPRi (dSaCas9-KOX1 + *CD5* sgRNAs). Control conditions omitting either AsCas12 crRNA or AsCas12 protein were included. The cells were analyzed by flow cytometry at the indicated time points post electroporation for percentage of cells positive for (A) CD5, (B) CD123, and (C) TCR. (D–F) MFI values of experiments described in (A)–(C). (G and H) Flow-cytometry plots are presented from the most efficient replicate 4 days post electroporation. The red gates indicate cells with the CD5^+^ CD123^−^ TCR^+^ phenotype for mock-treated cells, and the inverse CD5^−^ CD123^+^ TCR^−^ phenotype for CRISPR-treated cells. Percentages show percentage of parent gate. Data are presented as mean ± SEM (*n* = 3).

For complex genetic engineering, transient gene manipulation could be desirable in the context of simultaneous permanent knockout of a separate gene. Hence, we included Cas12a from *Acidaminococcus* (AsCas12a) for targeted knockout of the *TRAC* locus encoding the constant part of the T cell receptor (TCR) alpha chain. Importantly, Cas12a crRNAs do not complex with SpCas9 or SaCas9, or vice versa. This created a trimodal genetic engineering system using AsCas12a ribonucleoprotein (RNP) for *TRAC* knockout, dSpCas9-VPR mRNA for *CD123* upregulation, and dSaCas9-KOX1 mRNA for *CD5* repression. We used a previously reported AsCas12a crRNA for *TRAC* knockout by Cas12a RNP delivery.^28^ Jurkat cells were electroporated with dSpCas9-VPR and dSaCas9-KOX1 mRNA, and sgRNAs for both targets, as well as precomplexed Cas12a RNP consisting of recombinant Cas12a protein and *TRAC*-targeting crRNA (Figure 4). We observed that both *CD123* upregulation and *CD5* repression were efficient, peaking at 93% CD123^+^ cells and 80% CD5^−^ cells (Figures 4A, 4B, 4D, and 4E). For AsCas12a RNP delivery, we observed efficient *TRAC* knockout with average 67% TCR^−^ cells 4 days post electroporation (Figures 4C and 4F). Analysis of the CD5^−^ CD123^+^ population on day 4 showed that most cells were TCR^−^, confirming that the trimodal engineering had occurred in the same cells (Figures 4G and 4H). Overall, ∼65% of cells displayed the intended expression pattern for the three target genes (CD5^−^ CD123^+^, and TCR^−^) compared to 0.01% in the mock-treated population. This demonstrated efficient trimodal genetic engineering using three different Cas orthologs.

**Figure 5:**
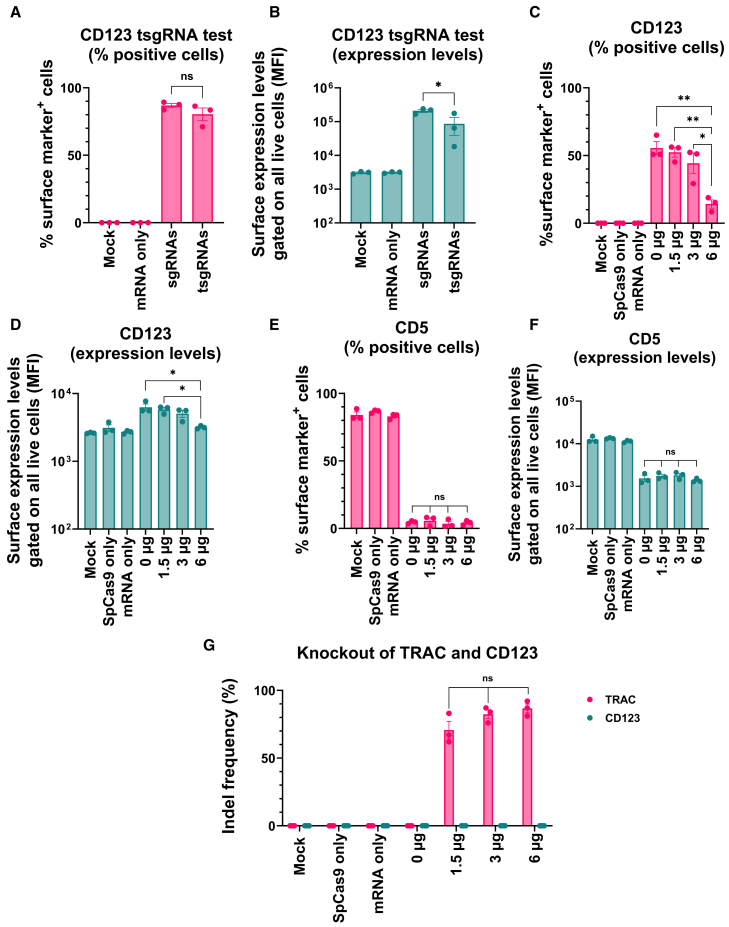
Simultaneous CRISPRa, CRISPRi, and gene knockout using truncated sgRNAs for CRISPRa is affected by sgRNA interference (A and B) Jurkat cells were electroporated with dSpCas9-VPR mRNA and either truncated sgRNAs (tsgRNAs) or standard-length sgRNAs targeting *CD123* (pool of four sgRNAs) for upregulation. Flow-cytometry analyses were performed 24 h after electroporation to determine (A) percentage of CD123^+^ cells and (B) median fluorescence intensity (MFI) of CD123 surface levels. (C–G) Cells were electroporated with SpCas9 protein and a full-length sgRNA (RNP) for knockout of the *TRAC* gene, dSpCas9-VPR mRNA and truncated sgRNAs (tsgRNAs) for *CD123* upregulation, and dSaCas9-KOX1 mRNA with full-length sgRNAs for repression of *CD5*. The amount of sgRNA:Cas9 RNP was varied (0–6 μg of Cas9 protein complexed with sgRNA at an sgRNA/Cas9 molar ratio of 2.5:1 and electroporated in a 20-μL electroporation volume). CD123 and CD5 expression were measured by flow cytometry 72 h post electroporation to analyze (C) percentage of CD123^+^ cells, (D) CD123 MFI, (E) percentage of CD5^+^ cells, and (F) CD5 MFI. (G) Indels generated at the *TRAC* and *CD123* loci were assessed by Sanger sequencing and ICE analysis 4 days post electroporation. Data are presented as mean ± SEM (*n* = 3). ns, not significant (*p* > 0.05); ∗*p* ≤ 0.05, ∗∗*p* ≤ 0.01. *p* values were calculated using a one-way ANOVA with Tukey’s multiple comparisons test.

Since AsCas12a-mediated knockout of the *TRAC* locus could be successfully implemented for trimodal genetic engineering, we next sought to simplify the system to use only two Cas orthologs. It has previously been established that Cas9 complexed with sgRNAs with truncated spacers (<16 nt) (tsgRNAs) still allows for localization of SpCas9:sgRNA RNP to the target sequence but does not support the formation of double-strand breaks by Cas9.^29^^,^^30^ Based on this, we first compared tsgRNAs to full-length sgRNAs for *CD123* CRISPRa, which showed a small decrease in CRISPRa activity with tsgRNAs (Figures 5A and 5B). We next performed combined delivery of SpCas9 recombinant protein complexed to a full-length sgRNA (SpCas9 RNP) for knockout of the *TRAC* locus, dSpCas9-VPR mRNA and tsgRNAs for *CD123* upregulation, and dSaCas9-KOX1 mRNA with full-length sgRNAs for repression of *CD5*. Reagents for all three targets genes were co-delivered into Jurkat cells while varying the SpCas9 RNP amounts from 0 to 6 μg (20 μL electroporation volume) using previously established Cas9 and sgRNA complexation ratios for *TRAC* RNP delivery (Figures 5C–5G).^31^ Flow-cytometry analysis of *CD5* and *CD123* expression 3 days post electroporation showed efficient *CD5* repression across all SpCas9 RNP amounts (Figures 5E and 5F), whereas activation of *CD123* was highly reduced when using the highest RNP amount, with only 14% of cells showing induced *CD123* expression (Figures 5C and 5D). Sequencing analysis of the *TRAC* and *CD123* loci showed high insertion or deletion (indel) frequencies at the *TRAC* locus, reaching almost 87% when using 6 μg of Cas9 protein and dropping to 71% when using 1.5 μg (Figure 5G). No indels were detected at the *CD123* locus, confirming that tsgRNAs do not support Cas9 DNA cleavage (Figure 5G).

**Figure 6:**
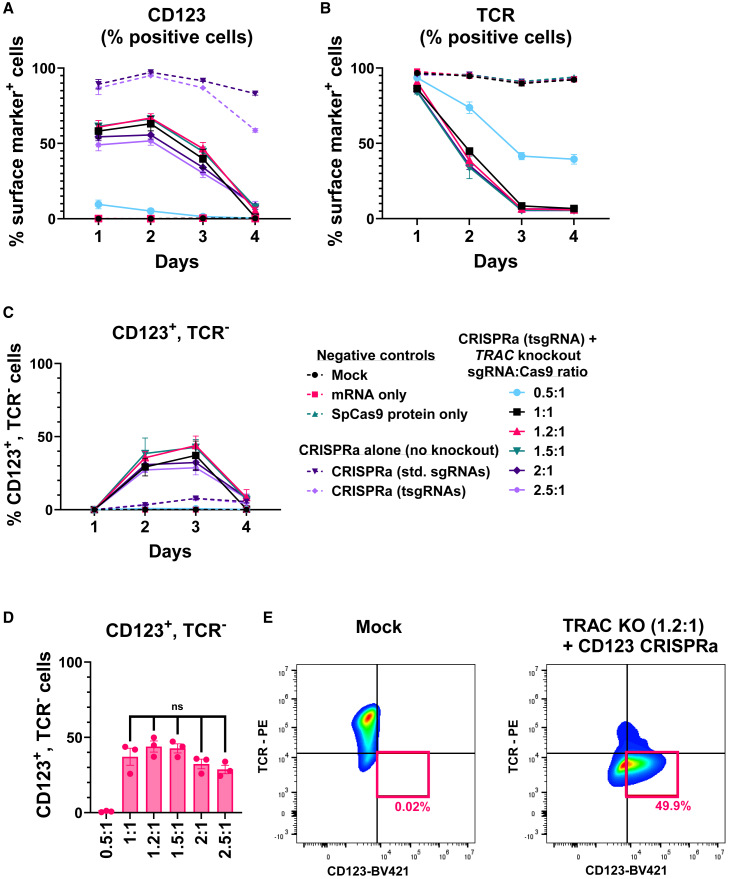
The sgRNA:protein complexation ratio used to form Cas9 RNP for *TRAC* knockout impacts the efficiency of *CD123* CRISPRa (A–C) Jurkat cells were electroporated with *TRAC*-targeting sgRNA:SpCas9 RNPs complexed at different ratios and dSpCas9-VPR mRNA and four modified sgRNAs targeting *CD123* for CRISPRa. The proportion of (A) CD123^+^ cells, (B) TCR^+^ cells, and (C) CD123^+^ TCR^−^ cells were analyzed by flow cytometry at the indicated time points post electroporation. (D) The percentage of CD123^+^ TCR^−^ cells at day 3 post electroporation from the experiment in (C). (E) Flow-cytometry plots from (A)–(C) at day 3 post electroporation shows CD123^+^ TCR^−^ cells for the two conditions (*n* = 3). The flow-cytometry plots are presented from the most efficient replicate. Data are presented as mean ± SEM (*n* = 3).

Since the *TRAC-*targeting SpCas9 RNP was complexed with a molar excess of sgRNA (2.5:1), we hypothesized that non-complexed *TRAC* sgRNA competed with *CD123* tsgRNAs during intracellular dSpCas9-VPR RNP formation, thereby effectively reducing the degree of *CD123* CRISPRa. Analogously, the sgRNAs might also compete for available Cas9 protein and reduce *TRAC* knockout. To confirm this hypothesis, we set up experiments testing simultaneous *TRAC* knockout and *CD123* CRISPRa at different ratios of *TRAC* sgRNA and Cas9 complexation (Figures 6 and S4). At the different ratios, we used a fixed amount of Cas9 protein (6 μg) and *CD123* activation, and *TRAC* knockout was measured over 4 days by flow-cytometry detection of the TCR.

When performing CRISPRa alone without *TRAC* RNP, high levels of *CD123* activation were observed, peaking at 95% CD123^+^ cells, but this activation declined faster for tsgRNAs than for standard sgRNAs (Figure 6A, light-purple vs. dark-purple dotted lines). When including *TRAC* RNP complexed at various sgRNA/Cas9 ratios, we found that the ratio highly impacted the degree of *CD123* activation. At low *TRAC* sgRNA amounts (0.5:1), the peak frequency of cells with CD123 activation was only 10% (Figure 6A, blue line), suggesting that free Cas9 protein could take up *CD123* tsgRNAs, thereby drastically reducing CRISPRa efficiency. *CD123* peak activation increased to 63%, 67%, and 66% of CD123^+^ cells when using the 1:1, 1.2:1, and 1.5:1 complexation ratio, respectively, whereas further increases in *TRAC* sgRNA amounts (2:1 and 2.5:1) decreased these frequencies to 56% and 52%, respectively.

*TRAC* knockout efficiencies measured by TCR expression were not compromised by gradually reducing *TRAC* sgRNA amounts from 2.5:1 to 1:1, but at 0.5:1 the frequency of TCR^+^ cells increased from 5% to 39% (Figure 6B). Analysis of the proportion CD123^+^ TCR^−^ cells showed that the optimal *TRAC* RNP complexation ratio was 1.2:1 or 1.5:1 (Figures 6C and 6D). Flow-cytometry analysis also confirmed that *TRAC* knockout and *CD123* activation occurred in the same cells (Figure 6E).

**Figure 7:**
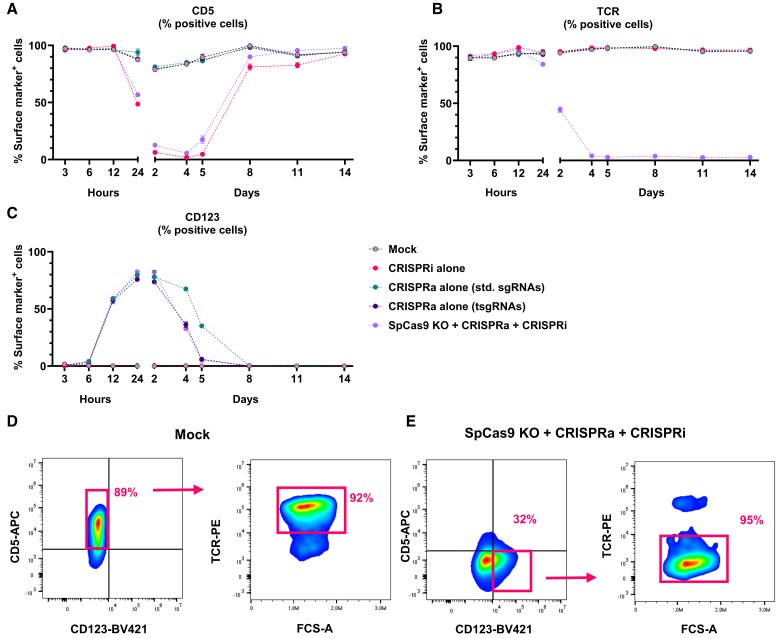
Simultaneous CRISPRa, CRISPRi, and knockout using two different Cas orthologs (A–C) Jurkat cells were electroporated with reagents for *TRAC* knockout (SpCas9KO), *CD123* CRISPRa (dSpCas9-VPR + truncated *CD123* sgRNAs), and *CD5* CRISPRi (dSaCas9-KOX1 + *CD5* sgRNAs). The cells were analyzed by flow cytometry at the indicated time points post electroporation for expression of (A) CD5, (B) CD123, and (C) TCR. (D and E) Flow-cytometry plots from the experiment in (A)–(C) at day 3 for (D) mock-treated cells showing CD5^+^ CD123^−^ TCR^+^ cells or (E) cells receiving all reagents showing CD5^−^ CD123^+^ TCR^−^ cells. Percentages show percentage of parent gate. The flow-cytometry plots are presented from the most efficient replicate. Data are presented as mean ± SEM (*n* = 3).

As both the 1.2:1 and 1.5:1 ratio proved optimal, we continued the study with the 1.2:1 ratio. We next used this ratio to test the efficiency and kinetics of orthogonal dSaCas-KOX1-mediated *CD5* CRISPRi, dSpCas9-VPR-mediated *CD123* CRISPRa, and SpCas9-based *TRAC* knockout in Jurkat cells (Figures 7 and S5). We found that *CD5* repression was almost complete and unaffected by simultaneous *CD123* CRISPRa and *TRAC* knockout (Figure 7A). *TRAC* knockout was also efficient, with 97% of cells being TCR^−^ (Figure 7B). *CD123* upregulation peaked at 82% CD123^+^ cells and was, in contrast to previous experiments (Figure 6), completely unaffected by including *CD5* CRISPRi and *TRAC* knockout (Figure 7C). The duration of *CD123* upregulation was still reduced when using tsgRNAs compared to full-length sgRNAs, narrowing the activity window of CRISPRa. This caused a reduction in the proportion of cells that were simultaneously CD5^−^ CD123^+^, so on day 4 post electroporation an average of 27% of cells were CD5^−^ CD123^+^ TCR^−^ (Figures 7D and 7E). This confirms that three modes of genetic engineering can be performed simultaneously in the same cells by combining two or three Cas orthologs, creating a mix-and-match system for gene editing and transcriptional modulation.

### Trimodal engineering of primary human T cells

To evaluate the activity of trimodal CRISPR-Cas engineering in human primary CD3^+^ T cells, we introduced dSpCas9-VPR mRNA for CRISPRa, dSaCas9-KOX1 mRNA for CRISPRi, and AsCas12a protein for gene knockout, alongside sgRNAs targeting *CD123* (activation), *CD5* (repression), and *TCR* (knockout). Initial results using the same sgRNA concentrations as in the equivalent experiment in Jurkat cells (shown in Figure 4) showed suboptimal downregulation of *CD5*, with an average of 77% of cells remaining CD5^+^ (Figure S6). This prompted us to investigate higher concentrations of sgRNAs for both dSaCas9-KOX1 and KOX1-dSpCas9 (Figure S7). KOX1-dSpCas9 with the normal sgRNA concentration (0.05 μg/μL of each sgRNA) effectively repressed *CD5*, with a mean of 17% CD5^+^ cells by day 3 post electroporation. Similar repression levels were observed at the higher sgRNA concentration, but this degree of repression was maintained for an additional 24 h compared to the standard sgRNA concentration. However, overall duration of repression was not impacted by sgRNA concentration. For dSaCas9-KOX1, consistent with our preliminary experiments, the use of standard sgRNA concentration in the dSaCas9-KOX1 system led to suboptimal CRISPRi efficiency, with the proportion of CD5^+^ cells reaching only 45%. In contrast, high sgRNA concentrations closely mirrored the efficiency of the dSpCas9 system at standard sgRNA concentration. These findings show different sgRNA concentration requirements for the two Cas orthologs in Jurkat cells vs. primary T cells but demonstrate that with optimization it is possible to achieve potent *CD5* downregulation in both cell types.

**Figure 8:**
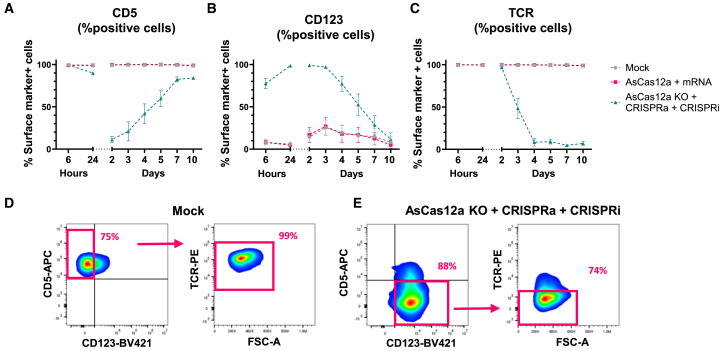
Combinatorial CRISPRa, CRISPRi, and knockout in primary human T cells (A–C) Primary human T cells were electroporated with reagents targeting *TRAC* for gene knockout using AsCas12a (KO), activation of *CD123* via CRISPRa (dSpCas9-VPR with *CD123* sgRNAs), and inhibition of *CD5* expression via CRISPRi (dSaCas9-KOX1 with *CD5* sgRNAs). Post electroporation, cells were analyzed by flow cytometry at various time points to assess the expression of (A) CD5, (B) CD123, and (C) TCR. (D and E) Flow-cytometry plots from day 3 show (D) mock-treated cells and (E) cells subjected to the complete trimodal engineering protocol. In (D) the CD5^+^ CD123^−^ TCR^+^ population is shown, while in panel (E) the CD5^−^ CD123^+^ TCR^−^ population is shown. The percentages indicate the proportion of cells of the parent gate within each parent population. The flow-cytometry plots are presented from the most efficient donor. Data were collected from three independent T cell donors (*n* = 3), with data points representing mean values ± SEM.

With optimized sgRNA concentrations for *CD5* CRISPRi with dSaCas9-KOX1, we applied these reagents to the trimodal system, combining *CD123* CRISPRa, *CD5* CRISPRi, and *TRAC* knockout (Figures 8 and S8). The results demonstrated simultaneous and efficient *CD5* repression, *CD123* activation, and *TRAC* knockout. *CD5* expression was completely repressed in approximately 89% of cells by day 2 post electroporation (Figure 8A), while *CD123* upregulation was evident as early as 6 h post electroporation, with 76% of cells expressing *CD123*. By days 1–3 *CD123* expression peaked, averaging 98% CD123^+^ cells, whereas mock-treated or mRNA-only-treated cells maintained non-detectable or low *CD123* expression, which has been documented previously (Figure 8B).^32^^,^^33^ Both *CD5* repression and *CD123* activation gradually diminished over time, with most cells returning to baseline levels by day 10 post electroporation. For AsCas12a RNP delivery, efficient and permanent *TRAC* knockout was achieved, averaging 92% TCR^−^ cells 4 days after electroporation (Figure 8C). Subgating on CD5^−^ CD123^+^ cells on day 3 showed potent *TRAC* knockout in this population, confirming that trimodal engineering occurred in the same cells (Figures 8D and 8E). Analysis of the CD5^−^ CD123^+^ TCR^−^ population showed that on average 44% of the cells expressed this phenotype. These data indicate successful application of the trimodal genetic engineering system in human primary T cells.

To evaluate any impact on T cell fitness of the trimodal CRISPR engineering, absolute T cell counts and viabilities were tracked throughout the experiment but did not show any significant toxicity or adverse impact on expansion (Figures S9A–S9C). We also evaluated whether the trimodal genetic engineering system affected the physiology of primary T cells. We first analyzed proportions and ratios of CD4 and CD8 T cells and found no difference between the groups (Figure S10A). Finally, we investigated cytokine production following T cell stimulation as a measure of immune cell functionality. On day 4 after electroporation, cells were stimulated with phorbol 12-myristate 13-acetate (PMA) and ionomycin for 5 h to activate signaling pathways independently of the TCR (Figure S10B). Levels of secreted tumor necrosis factor α (TNF-α) and interferon-γ (IFN-γ) protein measured by ELISA from non-stimulated cells were consistently low across all conditions, confirming that trimodal CRISPR engineering did not introduce overt dysfunction. PMA and ionomycin stimulation induced high expression of both cytokines, but a 1.7-fold reduced TNF-α secretion was observed in the trimodal CRISPR-treated cells compared to controls while IFN-γ secretion was unaffected. Combined, these findings suggest that delivery of trimodal engineering components mostly preserves basic T cell health and functionality.

## Discussion

In this study, we screen multiple RNA-delivered CRISPRa and CRISPRi transcriptional effectors. We find VPR to be the most potent transcriptional activator, in the context of both a direct Cas9 fusion and the SunTag system. While there was no difference between these two systems for two of the target genes, a marked difference was observed for *CD19*, where the direct fusion was superior and the SunTag effectors showed little to no activity. The large difference between target genes can likely be ascribed to differences in epigenetic status across the genes. We have previously observed target genes that were not amenable to CRISPRa by dSpCas9-VPR even though up to ten different sgRNAs were tested and all ten were functional in facilitating high levels of indels when using an active Cas9 nuclease, confirming successful RNP complex formation and localization to the target gene.^22^ Future investigations of the correlation between CRISPRa activity, epigenetic marks, and chromatin status of the target gene will be crucial to dissecting and potentially overcoming such CRISPRa limitations.

The screening of CRISPRi effectors yielded very similar potencies for the three repressors when fused to dSpCas9. On the other hand, when using dSaCas9, KOX1 was much more potent than the other two repressors when targeting *CD3e*. Such a difference could be ascribed to different binding strength and kinetics of dSaCas9 vs. dSpCas9, possibly affected using different protospacers for the two systems, but it could also be a consequence of reduced compatibility between dSaCas9 and the repressors. The constructs employed in this study used previously reported fusion protein configurations to the extent available, but fusion positions have generally not been exhaustively explored and might be subject to enhancement, as could NLS architecture. Prior studies using plasmid or lentiviral delivery have found KOX1-MeCP2 to be superior to KOX1 alone, but we observed the opposite in the present study.^9^^,^^27^ This might be due to the different delivery modalities with plasmid and lentiviral vectors supporting accumulation of protein levels over multiple days, and these delivery modalities might also be less susceptible to translational differences between mRNAs.

The use of two Cas9 orthologs facilitates an orthogonal CRISPRa/CRISPRi system where all components can be delivered simultaneously without sgRNA cross-complexation between Cas9 enzymes. A single delivery step simplifies experimental procedures and reduces toxicities observed with consecutive electroporations.^34^ Still, as observed in our orthogonal, multiplexed experiments, onset and kinetics of CRISPRa and CRISPRi are target and modality specific, which need to be considered for any application. We also observed a reduction in *CD3e* repression efficiency in the multiplex setting. This is interesting, as *CD3e* was also the only gene that displayed marked differences between the three different CRISPRi effectors for dSaCas9. Combined, this indicates that *CD3e* is more difficult to repress and therefore more susceptible to reagent limitations, which might be overcome by further optimizations of delivery conditions.

Several studies have demonstrated high specificity of CRISPRa and CRISPRi, mainly driven by use of specific sgRNAs, but also the requirement for localization around TSSs to observe functional off-target effects.^11^^,^^17^^,^^18^^,^^24^^,^^35^ Even in cases where off-target sites might overlap with a TSS region, the transient nature of CRISPRi and CRISPRa significantly reduces the potential for any sustained unintended consequences. Our off-target analyses also showed high specificity of orthogonal *CD123* CRISPRa and *CD5* CRISPRi, and no global effect on the transcriptome was observed following either *CD123* CRISPRa + *CD5* CRISPRi or by overexpression of dCas9-fused effectors compared to dCas9 alone. The few identified DEGs could not be attributed to unintended sgRNA binding around the TSS regions, but we cannot rule out any off-target effects from binding to distant regulatory elements such as enhancers and silencers. However, we find it more likely that the DEGs are instead attributable to downstream signaling events following *CD123* upregulation and/or *CD5* repression. For example, upregulation of *CD123*, the interleukin-3 (IL-3) receptor, is known to enhance T cell survival and proliferation. *CD5* downregulation is associated with altered TCR signaling, potentially affecting T cell activation thresholds and immune response modulation.^36^^,^^37^^,^^38^ Overall, our analyses do not invoke major concerns in relation to off-target effects and support the utility of these platforms for transcriptional modulation.

Trimodal engineering performing CRISPRa, CRISPRi, and gene knockout was investigated initially using AsCas12a-mediated knockout and then simplified to explore the performance of truncated SpCas9 sgRNAs for *CD123* CRISPRa, enabling the use of nuclease-proficient SpCas9 for *TRAC* knockout. We did not observe any nuclease activity at the *CD123* locus, confirming previous reports that sgRNAs with spacer sequences truncated to 14–16 nt support RNP complexation and protospacer binding while preventing Cas9 nuclease activity. However, we did observe a small decrease in CRISPRa efficiency when using truncated sgRNAs for the trimodal system. The duration of activation was also reduced, suggesting that the use of tsgRNAs for CRISPRa could be a general approach to tune efficiency and duration of CRISPRa. This adds a potential new layer of flexibility to the CRISPR transcriptional modulation toolbox. Importantly, the concept of using truncated sgRNAs for CRISPRa extends to SaCas9 and Cas9 from *Streptococcus thermophilus* (StCas9) and has also been demonstrated for CRISPRi.^30^^,^^39^

Not unexpectedly, we observed that dSpCas9-mediated *CD123* CRISPRa with RNA-delivered components was influenced by excess sgRNA from *TRAC* SpCas9 RNP formation. Conversely, at low *TRAC* sgRNA amounts non-complexed SpCas9 protein likely sponged *CD123* sgRNAs, explaining the observed reduction in *CD123* activation. Hence, a two-modality system relying on the same Cas ortholog is vulnerable to imbalances between unsaturated Cas9 enzymes and excess sgRNA. This is particularly the case for combined use of recombinant SpCas9 enzyme and mRNA-delivered SpCas9. Optimally, the simultaneous use of two different saturated RNPs complexed separately *in vitro* might constitute the ideal approach but is challenged by the lack of available recombinant dSpCas9-VPR protein. To our knowledge, the only CRISPRa effector produced as recombinant protein is dSpCas9-VP64. Furthermore, intracellular sgRNA exchange between SpCas9 enzymes might still reduce activities.^40^

To investigate applicability to primary cells, human primary T cells were subjected to trimodal engineering using the three different Cas orthologs. Initially, we observed differences in magnitude and duration of *CD5* repression between dSpCas9 and dSaCas9 that was dependent on sgRNA concentration (Figure S7). This was not observed in Jurkat cells, suggesting cell-type-dependent differences that might also extend across genetic loci. Hence, for each new target gene and cell type, a careful titration of reagents might be needed to reach optimal transcriptional modulation. Importantly, with optimized conditions we were able to demonstrate trimodal engineering efficiencies in primary T cells that were high and comparable to those observed in Jurkat cells. Crucially, this did not compromise key aspects of fundamental T cell physiology, such as viability, proliferation, CD4 and CD8 proportions, or cytokine secretion following stimulation. We did observe a notable reduction in TNF-α secretion, whereas IFN-γ levels remained unchanged. As this was an sgRNA-dependent effect, it is likely attributed to the specific genetic manipulations (*CD123* upregulation and/or *CD5*/*TCR* loss). As previously mentioned, *CD123* and *CD5* affect T cell biology, as both are implicated in regulating T cell signaling pathways, and, in fact, He et al. found lower proportions of TNF-α^+^ IFN-γ^+^ T cells with *CD5* knockout compared to controls following stimulation by dendritic cells.^33^^,^^41^^,^^42^

The trimodal engineering serves as a proof of concept of the versatility of orthogonal CRISPR systems by enabling three separate CRISPR-Cas-mediated functions to be exerted by three different Cas orthologs. This orthogonality might be extended to other genome-engineering tools such as the combinatorial use of epigenome editors, base editors, and prime editors. This could potentially also help circumvent challenges with protospacer adjacent motif (PAM) availability by adding more potential sites that can be targeted within the sgRNA design window for each gene.

As previously reported, the onset of CRISPR activation usually occurs within the first 12 h after electroporation, with CRISPR inhibition having a slower onset, usually within 2–4 days after electroporation.^22^ Our study showed that the kinetics of the activated target genes varied when using the same dSpCas9-effector construct, indicating that the observed target variability is not just an effect of the mechanism used by the activation domain but could indicate that the epigenetic landscape around the regulatory elements of the target gene, as well as turnover rate of mRNA and protein, could play a part in the effect and kinetics of CRISPR-based regulation. In addition, it appeared that the use of truncated sgRNAs for activation might be a viable way of affecting the kinetics without larger impact on the efficiency by shortening the sgRNAs. This highlights the importance of considering kinetics when it comes to designing CRISPRa and CRISPRi setups for new target genes. More studies are needed to determine to which extent the observed target variability is dependent on the recruitment mechanism of the activation domain and on the local chromatin context. The same is evident for CRISPR interference, for which the kinetics of repression are distinctly different from those of CRISPRa. When determining the effects of CRISPRi based on the protein expression levels, the effect is delayed in relation to CRISPRa, as the endogenous mRNA and protein will have to be cleared by the cell before any CRISPRi-mediated silencing can be detected by flow cytometry. This could account for the delayed onset of interference detected for the CRISPRi target genes. One potential challenge with implementation of orthogonal CRISPRa and CRISPRi could be the lack of overlapping activity windows depending on the target genes. For some uses, it might be crucial to induce the transcriptional changes simultaneously. We have largely observed transcriptional modulation that occurred with overlap but should it be needed, these systems might be tuned, for example by using attenuated systems employing mismatched sgRNAs or mutated sgRNA scaffolds.^43^^,^^44^

Orthogonal and multiplexed gene regulation by CRISPRa/CRISPRi could prove beneficial for transiently rewiring cellular pathways, changing surface expression of receptors, or modulating gene-expression profiles of signaling molecules. We have previously demonstrated the use of the RNA-delivered CRISPRa platform for transient activation of a silent reporter gene to enable immunomagnetic enrichment of gene-edited primary cells, and we have also manipulated differentiation trajectories of human hematopoietic stem cells and used CRISPRa to characterize genetic splice variants in easily accessible cells where the gene was otherwise not expressed.^22^^,^^45^^,^^46^ The combination of orthogonal transcriptional regulation systems with editing at the DNA level allows even more manipulation of the cells at once, with the choice between transient and permanent effects. We envisage that distinct phenotypic and functional cell states might be imposed by transient transcriptional regulation that leaves no DNA footprint, while gene editing could simultaneously endow the cells with permanently active synthetic biology such as expression of chimeric receptors. This could, for example, find use in the manufacturing of chimeric antigen receptor (CAR) T cells, where gene editing could provide cells with permanent expression of the CAR and loss of inhibitory receptors, while transient transcriptional manipulations might provide resistance to T cell differentiation and exhaustion related to the manufacturing process or even reprogram cells into the more potent T memory stem cell (T_SCM_) subset.^47^ We also foresee applications in genetic diseases and regenerative medicine, where permanent gene correction might be combined with transcriptional modulation of pathways that enhance transient processes such as cell homing, engraftment, and tissue regeneration. In conclusion, our studies show proof of concept for multilayered genetic perturbations that might accelerate the development of personalized regenerative treatments by modulating multiple gene networks simultaneously.

## Materials and methods

### Plasmid cloning

All plasmids were designed based on a backbone suitable for *in vitro* mRNA transcription previously described in Jensen et al.^22^ This plasmid contains a T7 promoter followed by a 45-bp 5′ UTR ending with a Kozak sequence after which the gene of interest (GOI) is inserted. The GOI is followed by a 93-bp 3′ UTR of the murine Hba-a1 gene and a 50-nt polyA tail and a unique restriction site for run-off *in vitro* transcription. The GOIs for CRISPRa experiments were as follows: dSpCas9-VPR from Jensen et al.,^22^ dSpCas9 for dSpCas9-VPRmini, and dSpCas9-p65p3-HSF1 amplified from the dSpCas9-VPR plasmid.^22^ VPRmini was amplified from Addgene plasmid 99698. pAAV-SCP1-dSa VPR mini.-2X snRP-1 BsaI gRNA was a gift from George Church (Addgene plasmid #99698; http://n2t.net/addgene:99698; RRID:Addgene_99698). sp65p3-HSF1 was amplified from Addgene plasmid 128136. pCE059-SiT-Cas12a-[Activ] was a gift from Randall Platt (Addgene plasmid #128136; http://n2t.net/addgene:128136; RRID:Addgene_128136). dSpCas9-SunTag_10× was amplified from Addgene plasmid 107310. dSV40-NLS-dCas9-HA-NLS-NLS-10xGCN4 was a gift from Hui Yang (Addgene plasmid #107310; http://n2t.net/addgene:107310; RRID:Addgene_107310). scFv-GCN4-VPR, scFv-GCN4-VPRmini, and scFv-GCN4-sp65p3-HSF1 were synthesized by Twist Bioscience. scFv-GCN4-VP64 was amplified from Addgene plasmid 60904. pHRdSV40-scFv-GCN4-sfGFP-VP64-GB1-NLS was a gift from Ron Vale (Addgene plasmid # 60904; http://n2t.net/addgene:60904; RRID:Addgene_60904). CRISPRi IVT template plasmids were produced as follows. KOX1-dSpCas9 was from Jensen et al.^22^ (as KRAB-dSpCas9) (Addgene plasmid #205248; http://n2t.net/addgene:205248; RRID:Addgene_205248). dSpCas9-KOX1-MeCP2 was amplified from Addgene plasmid 110821 (as dCas9-KRAB-MeCP2). dCas9-KRAB-MeCP2 was a gift from Alejandro Chavez and George Church (Addgene plasmid #110821; http://n2t.net/addgene:110821; RRID:Addgene_110821). ZIM3-dSpCas9 was amplified from Addgene plasmid 154472. pLX303-ZIM3-KRAB-dCas9 was a gift from Mikko Taipale (Addgene plasmid #154472; http://n2t.net/addgene:154472; RRID:Addgene_154472). dSaCas9-KOX1 was amplified from Addgene plasmid 106219. AAV CMV-dSaCas9-KRAB-bGHpA was a gift from Charles Gersbach (Addgene plasmid #106219; http://n2t.net/addgene:106219; RRID:Addgene_106219). dSaCas9 for dSaCas9-KOX1-MeCP2 and dSaCas9-ZIM3 was also amplified from Addgene plasmid 106219. dSpCas9 without effector fusion was amplified from the dSpCas9-VPR plasmid, and dSaCas9 without effector fusion was amplified from Addgene plasmid 106219. All plasmids and their respective sequences are listed in Table S1. Primers contained at least 30-bp compatible overhangs with the backbone for Gibson cloning. All primers were ordered from Sigma-Aldrich (Merck) and are listed in Table S2. Amplification of GOIs was performed using the Phusion Green Hot Start II High-Fidelity PCR Master Mix (Thermo Fisher Scientific) according to the manufacturer’s instructions. Non-template controls were included for all reactions. GOIs were purified from a 1% agarose gel using the GeneJet Gel Extraction Kit (Thermo Fisher Scientific) following the supplier’s instructions. The assembly of PCR fragments and linearized IVT vector were conducted using the NEBuilder HiFi DNA Assembly (New England BioLabs) using recommended ratios of insert to vector as well as incubation times provided by the supplier. The assembled products were then transformed into 10-beta Competent *E*. *coli* (New England BioLabs) and finally purified using NucleoBond Extra Midi EF (MACHEREY-NAGEL). All plasmid constructs were confirmed by Sanger sequencing (Eurofins Genomics and Macrogen) or whole-plasmid sequencing using Oxford Nanopore technology.

### *In vitro* transcription

IVT mRNA was generated as previously described by Jensen et al.^22^ with the following modifications. For the IVT reaction, uridine was substituted with N1-methyl-pseudouridine (Jena Bioscience) and with a CleanCap/GTP ratio of 0.8:1. The mRNA was purified and concentrated using the RNA Clean & Concentrator Kit (Zymo Research) according to the manufacturer’s manual. The mRNA quantity was verified using a DeNovix DS-11 Series spectrophotometer, and the RNA quality was verified on an Agilent 2100 Bioanalyzer (Agilent Technologies) using the RNA 6000 Nano Kit (Agilent Technologies) and accompanying protocol.

### sgRNAs

The sgRNAs for the *S*. *pyogenes* Cas9 system were ordered from Synthego as chemically modified sgRNAs containing 2′-*O*-methyl on the three terminal nucleotides at both ends and 3′-phosporothioate between the first three and last two bases.^21^ sgRNAs for the *S*. *aureus* Cas9 system were synthesized by either Synthego or SBS Genetech with 2′-*O*-methyl and 3′-phosphorothioate on the three terminal nucleotides at both ends. Spacer sequences are listed in Table S3. The sgRNA designs for CRISPRa and CRISPRi experiments were based on guidelines previously devised by Gilbert et al.^16^ and Sanson et al.^48^ and with at least 30 bp between adjacent protospacer sequences wherever possible due to PAM abundance. *TRAC* sgRNAs for SpCas9 was from Wiebking et al.,^31^ and *TRAC* crRNA for AsCas12a was from Kath et al.^28^

### Cell culture

Jurkat cells were cultured in RPMI-1640 medium supplemented with 5% heat-inactivated fetal calf serum (FCS), 2mM L-glutamine, 100 U/mL penicillin, and 100 μg/mL streptomycin. Peripheral blood mononuclear cells were isolated from de-identified buffy coats obtained from healthy adult donors from the Aarhus University Hospital Blood Bank by Ficoll-Plaque plus density gradient and from these, primary human T cells were purified by negative selection with the EasySep human T cell isolation kit (STEMCELL Technologies). The primary human T cells were cultured in X-VIVO 15 medium (Lonza) supplemented with 5% human albumin serum (Merck) and 10 ng/mL IL-7 and IL-2 (Peprotech). The cells were activated for 3 days with Dynabeads human T-activator CD3/CD28 (Thermo Fisher Scientific) at a 1:1 cell-to-bead ratio. All cells were counted on a Bio-Rad TC20 automated cell counter using trypan blue to exclude dead cells.

### Electroporation

All cells were electroporated using the 4D-nucleofector device from Lonza (X unit) in 20-μL-format Nucleocuvette strips. Cells were electroporated in the following electroporation buffers and programs. Jurkat cells: Opti-MEM (Thermo Fisher Scientific), CM138-P3; primary human T cells: solution 1 M, EO115-P3.^49^ For CRISPRa and CRISPRi singleplex RNA-based delivery experiments, unless otherwise specified, cells were electroporated with 0.095 μg/μL mRNA + 0.05 μg/μL of each of the sgRNAs. For orthogonal CRISPRa and CRISPRi experiments, cells were electroporated with 0.095 μg/μL dSpCas9-VPR mRNA along with 0.0125 μg/μL of each sgRNA for *CD123* (#1–4) and *NGFR* (#1–4), and 0.095 μg/μL dSaCas9-KOX1 mRNA along with 0.0167 μg/μL of each sgRNA for *CD5* (#1–3) and *CD3E* (#1–3). In primary human T cells for CRISPRa and CRISPRi experiments for trimodal engineering, at the optimized condition cells were electroporated with 0.095 μg/μL dSpCas9-VPR mRNA + 0.0125 μg/μL of each sgRNA for *CD123* (#1–4) and 0.095 μg/μL dSaCas9-KOX1 + 0.05 μg/μL of each sgRNA for *CD5* (#1–3).

For gene editing of *TRAC* with nuclease-active Cas9 protein (Alt-R S.p. Cas9 Nuclease V3; IDT), Cas9 and sgRNAs were incubated for 15 min at room temperature and later stored at 4°C prior to electroporation. RNP complexes were mixed with cells resuspended in 1 M electroporation buffer.^50^ Cas9 protein and sgRNAs were at a final concentration of 0.320 μg/μL and 0.160 μg/μL, respectively. Four days post electroporation, genomic DNA was extracted for analysis of indels using QuickExtract DNA extraction solution (Nordic Biolabs). The *TRAC* and *CD123* genomic region covering the sgRNA target site(s) were PCR amplified using the following primer pairs: (*TRAC*) Fw 5′-ATC ACG AGC AGC TGG TTT CT-3′, Rv 5′-CCC GTG TCA TTC TCT GGA CT-3′; (*CD123*) Fw 5′-ACT GTA ACC TCC TCC GCC TC -3′, Rv 5′-GAT ATC TTC CCG TGT GCG CT-3′. PCR products were either run on a 1% agarose gel and purified using the GeneJet Gel Extraction Kit (Thermo Fisher Scientific) or by using an enzyme-based PCR clean-up method. 10 μL of post-PCR reaction mixture was mixed with 7 μL of nuclease-free water, 0.5 μL of Exonuclease I (Thermo Fisher Scientific, #EN0581) for a final concentration of 0.556 U/μL, and 0.5 μL of FastAP thermosensitive alkaline phosphatase (Thermo Fisher Scientific, #EF0651) for a final concentration of 0,0278 U/μL. The reaction mixture was placed in a C1000 Thermal Cycler (Bio-Rad) running the following program: 37°C for 15 min and 80°C for 15 min. The purified PCR products were Sanger sequenced (Eurofins Genomics), and the sequencing files were analyzed by ICE CRISPR analysis tool (Synthego) to validate indel formations using a mock-electroporated sample as a wild-type control.

### Flow cytometry

Between 0.8 × 10^5^ and 3 × 10^5^ cells were collected and spun down at 300 × *g* for 5 min. Cells were washed in PBS and stained with a viability dye for 30 min to exclude dead cells from the analysis. The cells were then washed in PBS and resuspended in staining buffer (PBS, 2% FCS, 2 mM EDTA). Cells were stained with fluorochrome-conjugated antibodies (Table S4) in concentrations recommended by the supplier and incubated for 30 min. The cells were analyzed by flow cytometry on a NovoCyte Quanteon 4025 flow cytometer equipped with four lasers (405 nm, 488 nm, 561 nm, and 637 nm) and 25 fluorescence detectors (Agilent, Santa Clara, CA) or a CytoFLEX S flow cytometer equipped with four lasers (405 nm, 488 nm, 561 nm, and 638 nm) and 13 fluorescence detectors (Beckman Coulter, Brea, CA). All flow-cytometry experiments were individually compensated by individually stained compensation beads and analyzed in FlowJo (v.10.8.1). A representative gating scheme is shown in Figure S11. Surface-marker-positive cells were gated based on combinations of unstained, viability stain only, or target knockout. For counting human T cells to assess proliferation, the cells were counted on the flow cytometer using CountBright absolute counting beads (Invitrogen).

### RNA-seq analysis

Jurkat cells were electroporated in biological triplicates for each condition as described previously. The RNA amounts used for each condition in the electroporation mix were: (1) 0.095 μg/μL dSpCas9 + 0.095 μg/μL dSaCas9; (2) 0.095 μg/μL dSpCas9-VPR + 0.095 μg/μL dSaCas9-KOX1; and (3) 0.095 μg/μL dSpCas9-VPR + 0.095 μg/μL dSaCas9-KOX1 + 0.0125 μg/μL of each of the four sgRNAs against *CD123* and 0.0167 of each of the three sgRNAs targeting *CD5*. Three days following incubation the cells were lysed, and total RNA was purified using the ReliaPrep RNA Cell Miniprep system following the supplied protocol. The quality of the RNA was verified on a bioanalyzer and thereafter sent to Novogene for the RNA-seq workflow. Here, messenger RNA was purified from total RNA using poly-T oligo-attached magnetic beads. After fragmentation, the first-strand cDNA was synthesized using random hexamer primers followed by second-strand cDNA synthesis. The library was ready after end repair, A-tailing, adapter ligation, size selection, amplification, and purification. The library was checked with Qubit and real-time PCR for quantification and the bioanalyzer for size distribution detection. Quantified libraries were pooled and sequenced on an Illumina NovaSeq X Plus. A minimum of 63M raw reads were obtained from each sample. Raw sequences were screened using fastqc (v.0.12.1) to ensure high sequence quality. Reads were aligned to Genome Reference Consortium Human Build 38 patch release 14 (GRCh38.p14) using HISAT2 (v.2.2.1). Quantification of transcripts was performed using Subread (v.2.0.6). After quantification, the data were filtered to remove low-count features. We retained only those genes for which at least one sample had a read count greater than 5. DEGs were determined using DESeq2 (v.1.40.2), where statistical significance is classified by a *p* value of ≤0.05 and a log_2_ fold change of >2 or <−2 (Table S5). All the computing for this project was performed on the GenomeDK cluster. For off-target analysis, each of the four CD123 SpCas9 sgRNAs used for CRISPRa and each of the three CD5 SaCas9 sgRNAs used for CRISPRi were analyzed for potential off-targets in the genome using COSMID and CRISPOR (Table S5). The protospacer sequences were inputted, and the default parameters were used. For COSMID, this allows up to two mismatches between the sgRNA and the protospacer or 1-bp bulge in either the protospacer or the sgRNA. For SaCas9 PAM, the more relaxed 5′-NNGRRN-3′ PAM was used. For CRISPOR, up to four mismatches were allowed by default. Here, the canonical SaCas9 PAM 5′-NNGRRT-3′ was used. The identified potential off-target sites were then checked to see whether they were situated within a window of 10,000 bp of the TSS of any of the DEGs. TSSs were extracted using BioMart in Ensembl except for non-coding RNAs, in which case TSSs were manually extracted using NCBI.

### Cell stimulation and ELISA

Four days after electroporation with the trimodal CRISPR system, 500,000 primary human T cells from each donor were seeded into 96-well plates. Cells were stimulated with 25 ng/mL PMA and 1 μg/mL ionomycin for a duration of 5 h to induce cytokine production. Post stimulation, cell-culture supernatants were collected for cytokine quantification. TNF-α and IFN-γ concentrations were measured using the ELISA Max Deluxe Human TNF-α and IFN-γ kits, respectively (BioLegend). The experiment was conducted in technical duplicates or triplicates for each of the three T cell donors. Cytokine concentrations were interpolated from a standard curve, and supernatants were serially diluted to fit the assay’s dynamic range.

### Statistics

All statistical analyses were performed in GraphPad Prism (v.10.2.0). Statistical parameters are reported in the figure legends. Expression analyses were performed in biological triplicates unless otherwise specified. The significance of differences in multiple groups was determined by one-way or two-way ANOVA with Tukey’s multiple comparisons test. *p* values of <0.05 were considered statistically significant. In the figures, ns denotes no significance (*p* ≥ 0.05) and asterisks indicate ∗*p* ≤ 0.05, ∗∗*p* ≤ 0.01, ∗∗∗*p* ≤ 0.001, and ∗∗∗∗*p* ≤ 0.0001.

## Data and code availability

Source data for all graphs and raw RNA-seq files associated with this study have been deposited in the DRYAD repository. DRYAD: https://doi.org/10.5061/dryad.r4xgxd2q0. For additional information or to request reagents, please contact the corresponding author.

## Acknowledgments

We thank Pernille Thornild Møller for technical assistance. Some flow cytometry experiments related to Figure 1 were performed at the FACS Core Facility, Aarhus University, Denmark. We would like to thank the Bioinformatics Core Facility at Aarhus University, Department of Biomedicine, and especially Jacob Egemose Høgfeldt who helped with the full analysis of the RNA-seq data. The Bak Lab gratefully acknowledges funding support from grants from the EU Commission in the form of an ERC starting grant (HSC-CRISPR, project 101041231, Horizon Europe Pillar I) and a grant from the Horizon Research and Innovation Actions (project 101057438, Horizon Europe Pillar II). Views and opinions expressed are those of the authors only and do not necessarily reflect those of the European Union or the European Health and Digital Executive Agency (HADEA). Neither the European Union nor the granting authority can be held responsible for them. The Bak Lab also gratefully acknowledges grants from the 10.13039/501100003554Lundbeck Foundation Fellowship (R238-2016-3349), the Independent Research Fund Denmark (0134-00113B, 0242-00009B, and 9144-00001B), the 10.13039/501100009708Novo Nordisk Foundation (NNF19OC0058238 and NNF23OC0085659), 10.13039/100012774Innovation Fund Denmark (8056-00010B), the Danish Health Authorities (SST) (4-1612-391/1), the 10.13039/501100002808Carlsberg Foundation (CF20-0424), the Agnes and Poul Friis’ Foundation, and a Genome Engineer Innovation grant from Synthego.

## Author contributions

A.D.B., L.B., T.I.J., and R.O.B. conceived the study. A.D.B., L.B., S.F., and R.O.B. designed the experiments. A.D.B., L.B., and S.F. performed experiments and analyses with assistance from T.I.J. and K.M. All experiments were supervised by R.O.B. A.D.B., L.B., and S.F. wrote the manuscript with input from all the co-authors. All authors reviewed and contributed to editing the final manuscript.

## Declaration of interests

R.O.B. holds equity in UNIKUM Tx and is a consultant to UNIKUM Tx. T.I.J. is an employee of and holds equity in UNIKUM Tx. R.O.B. is inventor on patents or patent applications related to CRISPR-Cas and engineering of cellular products. R.O.B. reports research funding from Novo Nordisk.

## Supplemental information

Document S1. Figures S1–S11 and Tables S2–S4

Table S1. Plasma sequences

Table S5. RNA-sequencing data: DEG list, predicted off-target sites, TSS coordinates, and output from search within 10 kb

Document S2. Article plus supplemental information
