# Supplementary material for: Orthogonal transcriptional modulation and gene editing using multiple CRISPR-Cas systems
Source: Mol Ther. 2024 Nov 19;33(1):71–89. doi: 10.1016/j.ymthe.2024.11.024 (PMC11764084; doi:10.1016/j.ymthe.2024.11.024)
Supplement: Document S1. Figures S1–S11 and Tables S2–S4 [file mmc1.pdf]

YMTHE, Volume *33*

Supplemental Information

Orthogonal transcriptional modulation and gene editing using multiple CRISPR-Cas systems

Amalie Dyrelund Broksø, Louise Bendixen, Simon Fammé, Kasper Mikkelsen, Trine Ilsø Jensen, and Rasmus O. Bak

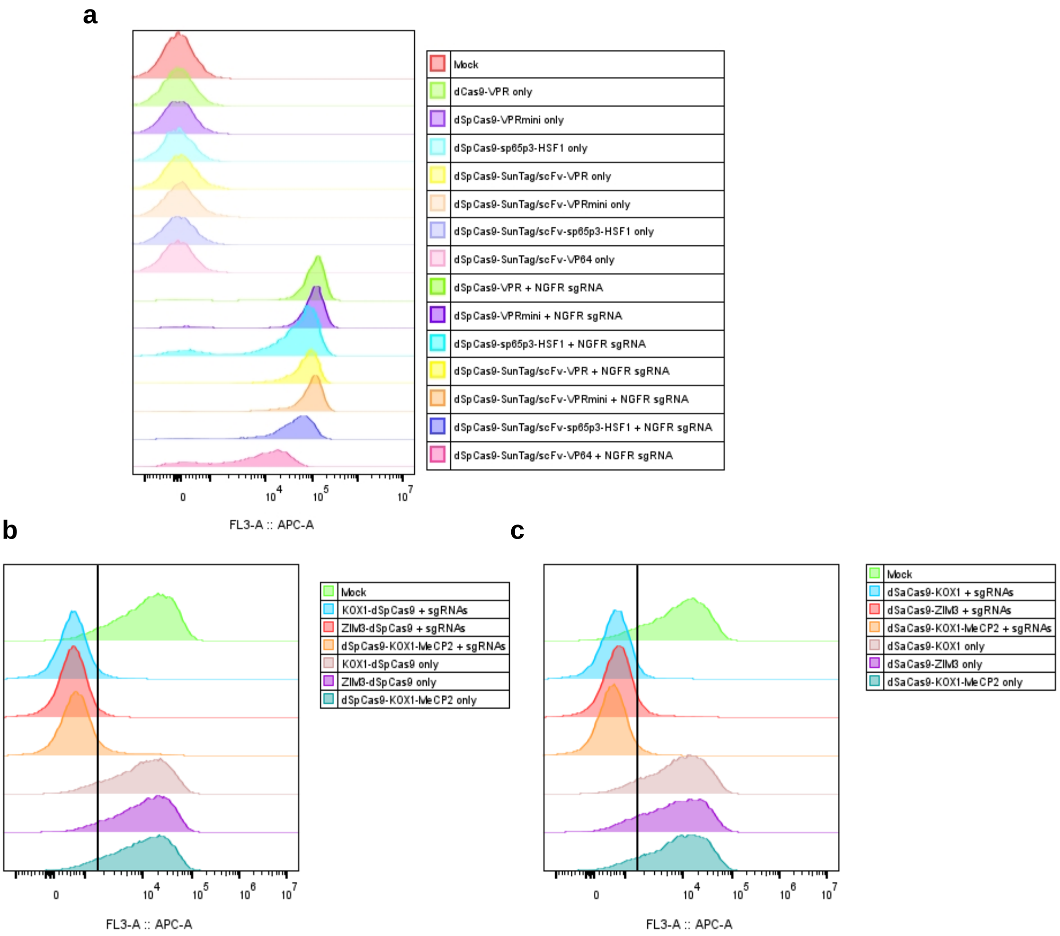

Figure S1: Representative flow cytometry histograms for *NGFR* upregulation and *CD5* repression. (a) Representative flow cytometry histograms from data presented in Figure 1c showing *NGFR* expression 24 hours post electroporation with CRISPRa reagents. **(b-c)** Representative flow cytometry histograms from data presented in Figure 2d and 2e, showing CD5 expression 72 hours post electroporation with Sp **(b)** and Sa **(c)** CRISPRi reagents.

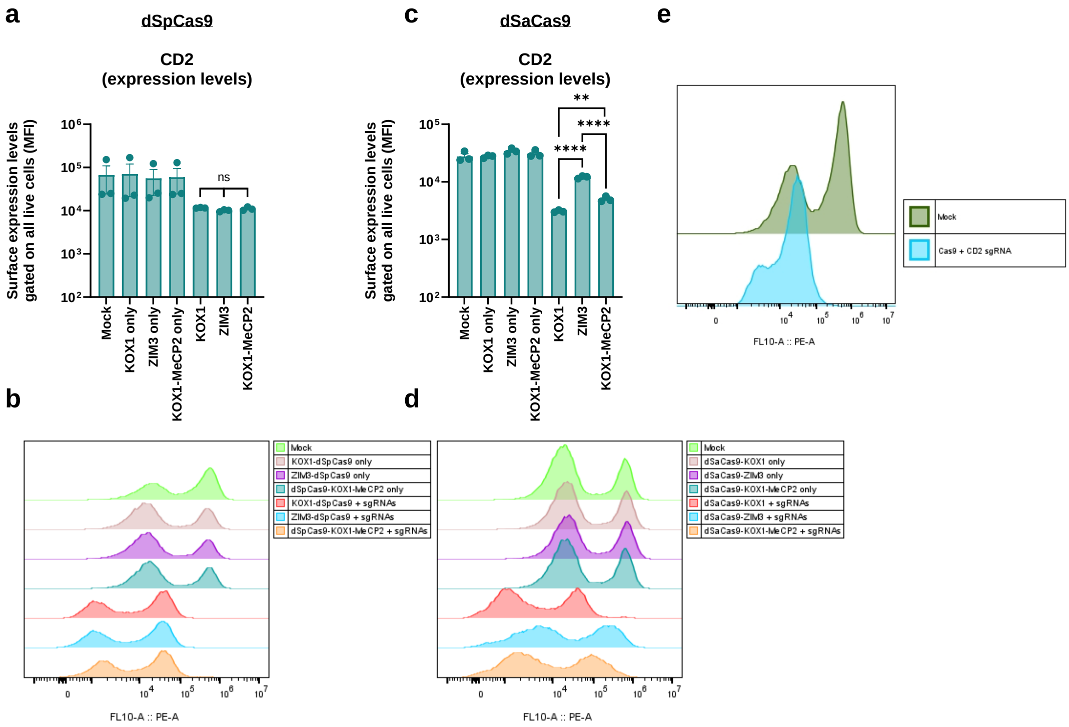

Figure S2: Downregulation of CD2 by dSpCas9- or dSaCas9-mediated CRISPRi. (a-b) Median fluorescence intensity (MFI) and representative flow cytometry histograms of *CD2* expression 72h post electroporation with IVT mRNA for the selected dSpCas9 CRISPRi systems and three chemically modified sgRNAs targeting the TSS of *CD2*. Mock refers to electroporation without IVT mRNA and modified sgRNAs. Effector only refers to electroporation with only the selected CRISPRi IVT mRNA without sgRNAs. **(c-d)** Same as (a-b) but for dSaCas9 constructs. **(e)** Flow cytometry histogram showing *CD2* expression in an untreated, electroporated Mock sample vs. an SpCas9-mediated *CD2* knockout. The *CD2* KO sample was to place the gate to distinguish $CD2^-$ and $CD2^+$ cells, but the KO sample also displayed two distinct CD2 populations. ns – nonsignificant ($P > 0.05$), * - $P \le 0.05$, ** - $P \le 0.01$, *** - $P \le 0.001$, **** - $P \le 0.0001$. P values were calculated using a one-way ANOVA with Tukey's multiple comparison's test, (n=3).

a

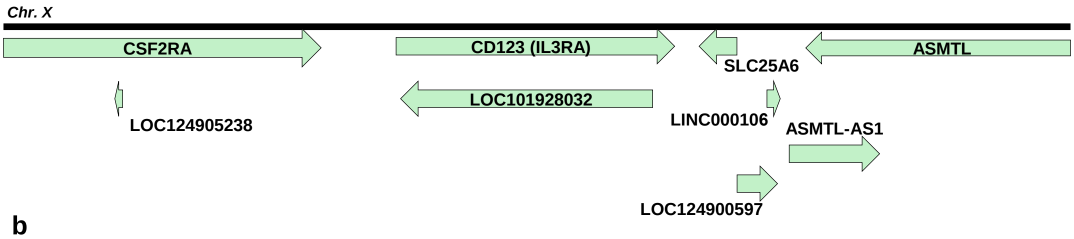

b

	dSpCas9 + dSaCas9 mRNA only			dSpCas9-VPR + dSaCas9-KOX1 mRNA only			CD123 CRISPRa + CD5 CRISPRi		
	Repl. #1	Repl. #2	Repl. #3	Repl. #1	Repl. #2	Repl. #3	Repl. #1	Repl. #2	Repl. #3
CSF2RA	0,000	0,000	0,000	0,021	0,000	0,000	0,000	0,027	0,000
LOC124905238	0,000	0,000	0,000	0,000	0,000	0,000	0,000	0,000	0,000
IL3RA	0,000	0,051	0,169	0,100	0,000	0,143	2,753	11,916	4,877
LOC101928032	0,018	0,016	0,051	0,000	0,021	0,054	0,306	0,154	0,061
SLC25A6	9,133	7,579	8,040	7,026	7,588	8,861	7,495	8,917	8,418
LOC124900597	0,550	0,339	1,503	0,725	0,367	0,472	0,355	0,251	0,531
LINC00106	3,699	2,506	2,995	3,991	3,082	3,018	0,894	1,970	3,348
ASMTL-AS1	0,632	0,804	1,792	0,642	0,893	1,100	0,638	1,453	0,675
ASMTL	11,456	4,768	0,817	4,373	4,501	6,744	5,476	4,363	7,898

Figure S3: Genomic locus containing the *CD123* gene (*IL3RA*) and neighboring genes (a) Schematic depiction of the region of the X chromosome containing the *CD123* gene (IL3RA) and the neighboring genes. **(c)** Transcript levels (TPM values) of *IL3RA* and neighboring genes from RNA-seq following the different treatments.

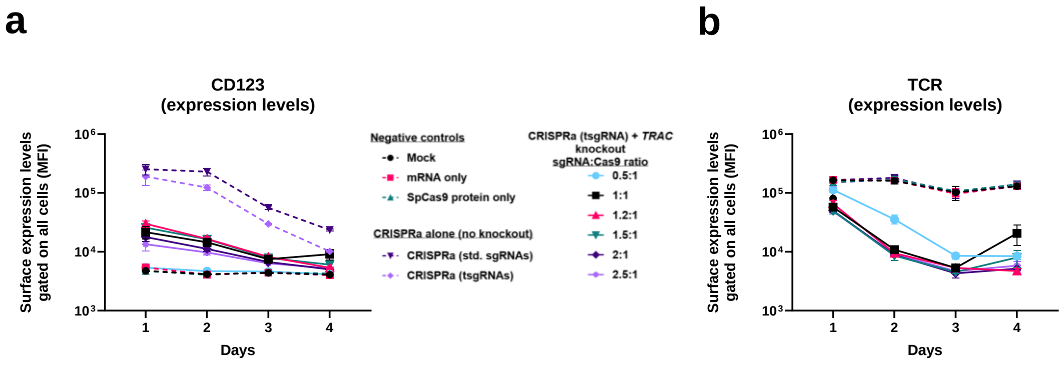

Figure S4: The sgRNA:protein complexation ratio used to form Cas9 RNP for *TRAC* knockout impacts the efficiency of *CD123* CRISPRa (a-b) MFI values from data shown in Figure 6. Jurkat cells were electroporated with *TRAC*-targeting sgRNA:SpCas9 RNPs complexed at different ratios and dSpCas9-VPR mRNA and four modified sgRNAs targeting *CD123* for CRISPRa. CD123 **(a)** and TCR **(b)** expression levels (MFI) were determined by flow cytometry at the indicated timepoints, (n=3).

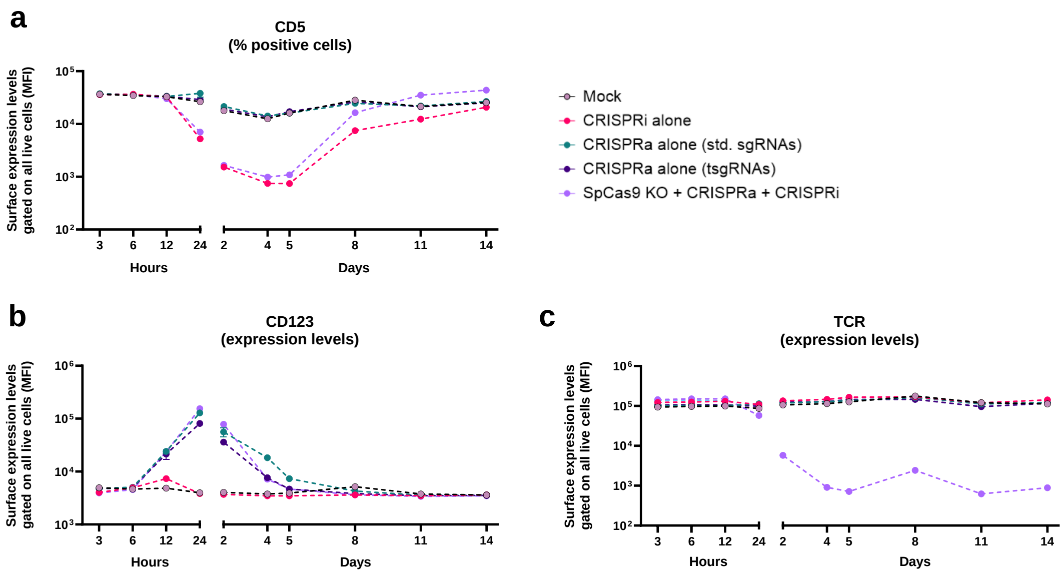

Figure S5: Simultaneous CRISPRa, CRISPRi, and knockout using two different Cas orthologs (a-c) MFI values from data shown in Figure 7a-c. Jurkat cells were electroporated with reagents for *TRAC* knockout (SpCas9 KO), *CD123* CRISPRa (dSpCas9-VPR + truncated CD123 sgRNAs), and *CD5* CRISPRi (dSaCas9-KOX1 + CD5 sgRNAs). The cells were analyzed by flow cytometry at the indicated time points post electroporation for MFI of **(a)** CD5, **(b)** CD123, and **(c)** TCR, (n = 3).

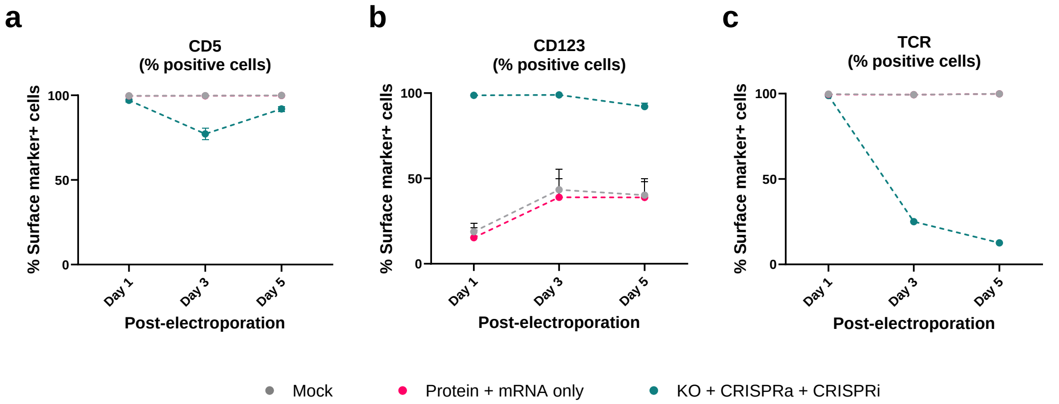

Figure S6: Trimodal genetic engineering in human primary T cells with suboptimal sgRNA amounts for dSaCas9-KOX1. Human primary T cells were electroporated with AsCas12a protein and *TRAC* sgRNA for KO, dSpCas9-VPR and *CD123* sgRNA for activation, and dSaCas9-KOX1 and *CD5* sgRNA for repression. The sgRNA concentration is 0.05 µg/µL of all sgRNAs combined (also referred to as std. concentration). On day 1, 3, and 5 post-electroporation, cells were analyzed by flow cytometry to assess the expression of **(a)** CD5, **(b)** CD123, and **(c)** TCR. Data were collected from three independent T cell donors, with data points representing mean values ± SEM, (n=3).

CD5 CRISPRi with different sgRNA concentrations

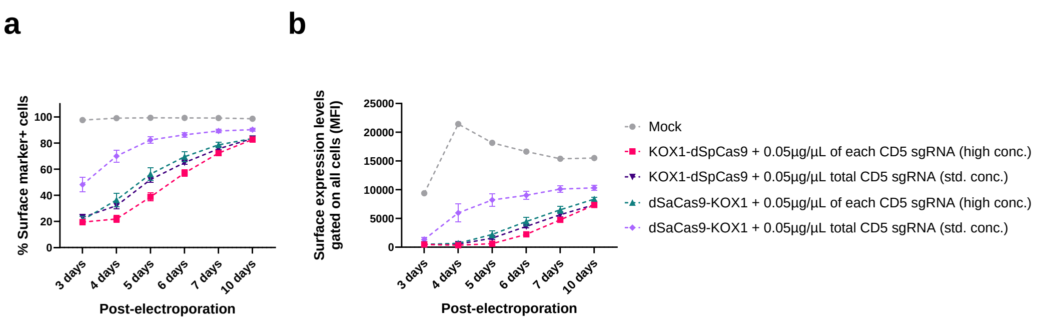

Figure S7: Kinetics of CRISPRi-mediated CD5 suppression using different Cas9 variants and different sgRNA concentrations. Human primary T cells were electroporated with mRNA encoding KOX1-dSpCas9 or dSaCas9-KOX1, alongside two different amounts of *CD5*-targeting sgRNAs; 0.05µg/µL of each sgRNA or 0.05µg/µL of total sgRNA **(a)** The proportion of $CD5^{+}$ cells and **(b)** the MFI of CD5 expression were measured at specified time points post-electroporation by flow cytometry. Each graph reflects data from one T cell donor performed in biological triplicates with data points indicating the mean ± SEM, (n=3).

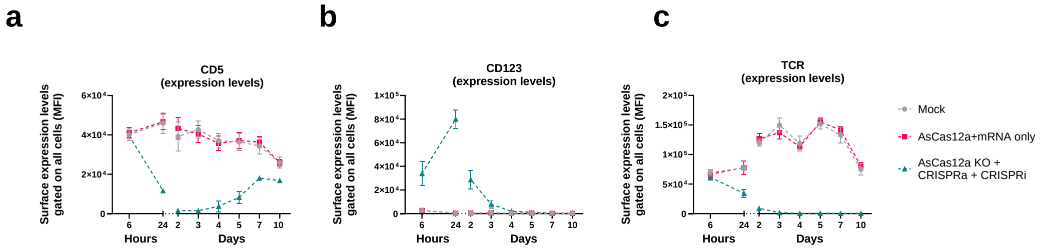

Figure S8: Combinatorial CRISPRa, CRISPRi, and knockout in primary human T cells. (a-c) MFI values corresponding to data presented in Figure 8a-c. Human primary T cells were electroporated for *TRAC* knockout (AsCas12a), *CD123* activation (dSpCas9-VPR + CD123 sgRNAs), and *CD5* inhibition (dSaCas9-KOX1 + CD5 sgRNAs). MFI for CD5 **(a)**, CD123 **(b)**, and TCR **(c)** were measured by flow cytometry at the indicated post-electroporation time points. Data points represent the mean ± SEM from three independent T cell donors, (n=3).

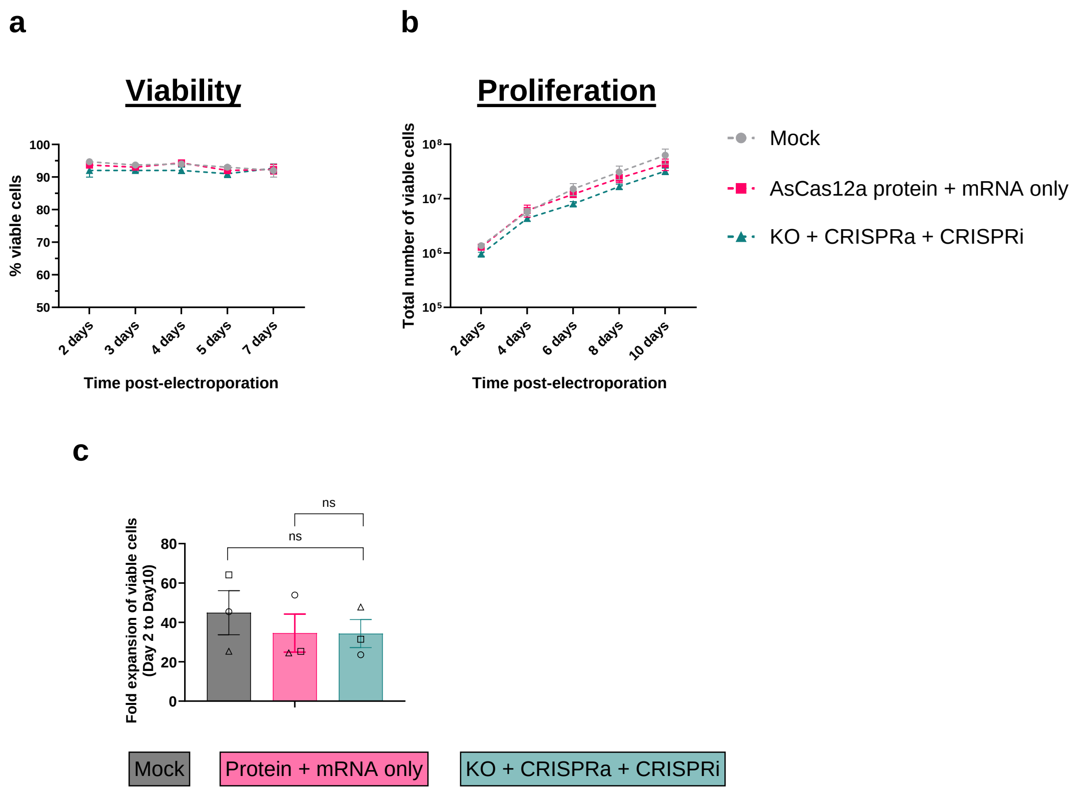

Figure S9: Viability and proliferation of T cells following trimodal CRISPR/Cas engineering. (a) Percent viability of T cells was measured at multiple time points post-electroporation using an automated cell counter and trypan blue for excluding dead cells. **(b)** Proliferation of viable T cells was monitored for 10 days after electroporation. The cells were counted on a flow cytometer using counting beads. **(c)** Fold expansion of T cells from day 2 to day 10. Data points represent mean ± SEM from three independent T cell donors, n=3, each shown with distinct symbols. Statistical significance was determined using one-way ANOVA with Tukey's multiple comparisons test ($P \geq 0.05$; ns, not significant).

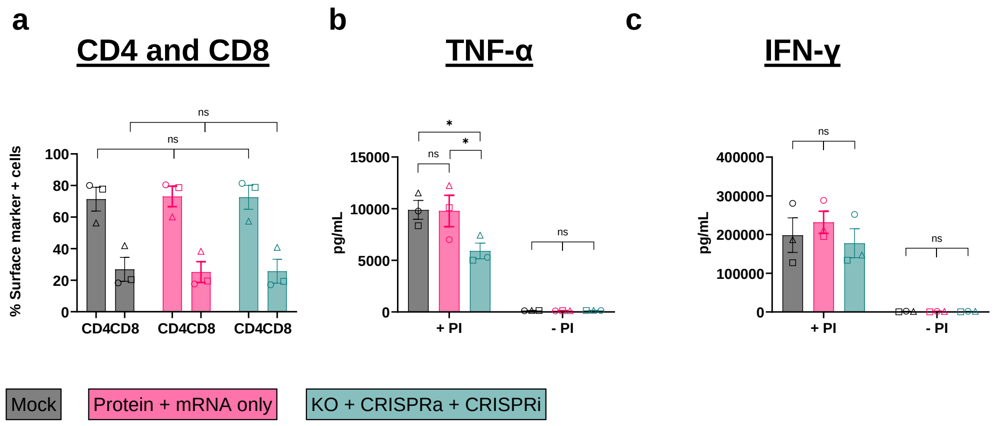

Figure S10: Investigating the impact of trimodal CRISPR/Cas engineering on T cell physiology. (a) The proportion of $CD4^+$ and $CD8^+$ cells was measured by flow cytometry analysis 4 days post-electroporation. **(b-c)** Levels of TNF-α **(b)** and IFN-γ **(c)** at day 4 post-electroporation secreted after 5-hours of stimulation with PMA and Ionomycin (+PI) (25 ng/mL PMA and 1 µg/mL ionomycin). Non-stimulated controls were included (-PI). Data points represent mean ± SEM from three independent T cell donors, n=3, each shown with distinct symbols. Statistical significance was determined using two-way ANOVA with Tukey's multiple comparisons test (*$P < 0.05$, ns: not significant).

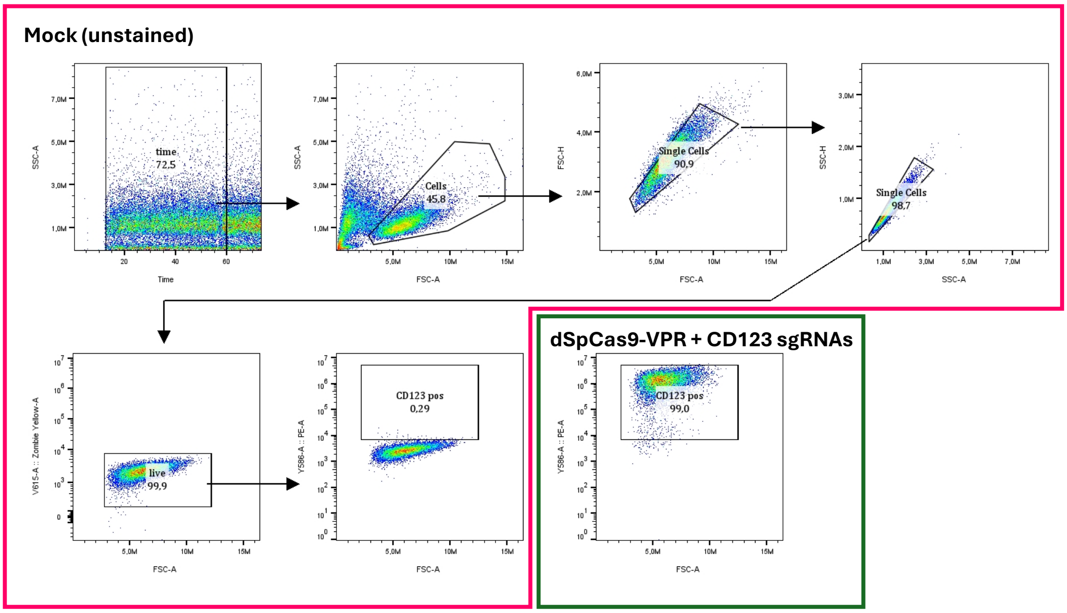

Figure S11: Representative gating strategy for flow cytometry detection of target gene expression. Representative gating strategy based on an unstained mock sample from Figure 1 (CD123 expression)(pink box). Gating was first performed on a time plot, then debris was gated out based on forward and side scatter. Afterwards, doublets were gated out, first based on forward scatter, then on side scatter. The live cells were determined by amine dyes, here ZombieYellow, and finally, target gene surface expression was determined (here antiCD123 antibody, PE). A sample treated with dSpCas9-VPR + *CD123* sgRNAs shows the gating on a CD123-upregulated sample (green box).

Table S1

Plasmid sequences

Go to the excel-file *"Supplemental table 1"*

Table S2

Oligonucleotide sequences

Name	Sequence (5'-3')
dSpCas9-VPRmini Gibson (fw)	ACGCGTTGATATCAACAAGTTTGTACAAAAAAGCAGGCTACAAAgaggccagcggttccg
dSpCas9-VPRmini Gibson (rv)	cccgcagaaggcagcttaattaagcggccgcctaaaacagagatgtgtcgaagatgg
dSpCas9-sp65p$_3$-HSF1 Gibson (fw)	GCGTTGATATCAACAAGTTTGTACAAAAAAGCAGGCTACAAAagcgggttgcctaatggg
dSp-sp65p$_3$ Gibson (rv)	gcaagccccgcagaaggcagcttaattaagcggccgcttaggcatagtcggggacatcat
dSpCas9-Suntag Gibson (fw)	gagagaaaagaagagtaagaagaaatatagctagcgccaccatggacaagaagta
margidSpCas9-Suntag Gibson (rv)	gcagaaggcagcttaattaagcggccgcttagaccttccttttcttttttggac
scFv-GCN4-sfGFP-VPR/VPRmini/sp65px3-HSF1 Gibson (fw)	cggtacctaatacgactcac
scFv-GCN4-sfGFP-VPR/VPRmini/sp65px3-HSF1 Gibson Gibson (rv)	aagggagagaagaagggc
scFv-GCN4-sfGFP-VP64 Gibson (fw)	gagagaaaagaagagtaagaagaaatatagctagcgccaccatgggccccgacat

scFv-GCN4-sfGFP-VP64 Gibson (rv)	agccccgcagaaggcagcttaattaagcggccgcttacggccatggcgtaggcg
dSpCas9-KRAB-MeCP2 Gibson (fw)	gagagaaaagaagagtaagaagaaatataGGCCACCatggacaagaagtactccattggg
dSpCas9-KRAB-MeCP2 Gibson (rv)	gcaagccccgcagaaggcagcttaattaagcggccgcCtgagactctctcagtcacgggt
ZIM3-dSpCas9 Gibson (fw)	gaaggcaagccccgcagaaggcagcttaattaagccactttccgctttttcttaggatct
ZIM3-dSpCas9 Gibson (rv)	agagagaaaagaagagtaagaagaaatataggccaccatgaacaattcccagggaagagt
dSaCas9-KRAB-MeCP2 Gibson (fw)	aaaaaggccggccaggcaaaaaagaaaaagggatccagaacactggttacgttcaaggac
dSaCas9-KRAB-MeCP2 Gibson (rv)	ggcaagccccgcagaaggcagcttaattaagcggccgctgagactctctcagtcacgggt
ZIM3-dSaCas9 Gibson (fw)	aggcaagccccgcagaaggcagcttaattaagcggccgccaccttgcgcttcttcttggg
ZIM3-dSaCas9 Gibson (rv)	aaaaaggccggccaggcaaaaaagaaaaagggatccgtgaccttcgaggatgtcactgtg

Table S3

List of chemical modified sgRNAs/crRNA.

Name	Sequence (5’-3’)	Cas orthologue	Supplier
CD123 #1<br>CD123 #2<br>CD123 #3<br>CD123 #4	CUGGCUAUACAGGCAGGGUU<br>GGCGUGAACGCUGGUGUCCA<br>UGGGAAACAGCACCCAGACA<br>GUUACGAAGCUGCUGACCCC	SpCas9	Synthego
tCD123 #1<br>tCD123 #2<br>tCD123 #3<br>tCD123 #4	CUGGCUAUACAGGCA<br>GGCGUGAACGCUGGU<br>UGGGAAACAGCACCC<br>GUUACGAAGCUGCUG	SpCas9	Synthego
CD271 #1<br>CD271 #2<br>CD271 #3<br>CD271 #4	GCGGGCGGGCGCGGUUCCGG<br>GAGAAGCGCAGCGGGGUGCG<br>CGGGAACUGGGUACCAGGGC<br>GGACCAGGGCGUCCCCACUG	SpCas9	Synthego
CD19 #1<br>CD19 #2<br>CD19 #3<br>CD19 #4	CCACCGCCUUCCUCUCUGGG<br>GGAGGCAAGUGUUGUGAGUC<br>UUUCCCGUGGUAGUGAGAGC<br>UCAACCAUGGGUGUCUGCGG	SpCas9	Synthego
CD5 #1<br>CD5 #2<br>CD5 #3<br>CD5 #4	GGCCAGAAACCAUGCCCAUG<br>CAGCUGGGCACCGCAGGUGA<br>AGCGGUUGCAGAGACCCCAU<br>GGGUGGGACAUGCAGGACAG	SpCas9	Synthego
CD5 #1	AGAAGGCCAGAAACCAUGCCCGUU<br>UUAGUACUCUGGAAACAGAAUCUA<br>CUAAAACAAGGCAAAAUGCCGUGU<br>UUAUCUCGUCAACUUGUUGGCGAG<br>AUUUU	SaCas9	SBS Genetech<br><br><br>SBS Genetech
CD5 #2	GGGCAGCUGGGCACCGCAGGUGU<br>UUUAGUACUCUGGAAACAGAAUCU<br>ACUAAAACAAGGCAAAAUGCCGUG<br>UUUAUCUCGUCAACUUGUUGGCGA<br>GAUUUU		SBS Genetech
CD5 #3	GGGCUCGCUCCAGUGCAAGGAGUU<br>UUAGUACUCUGGAAACAGAAUCUA<br>CUAAAACAAGGCAAAAUGCCGUGU<br>UUAUCUCGUCAACUUGUUGGCGAG<br>AUUUU		
CD2 #1	GaagagcUcagaaUcaaaag	SpCas9	Synthego

CD2 #2<br>CD2 #3	UUacaUggaaagcUcaUcUU<br>UcUgaUUUUcaaUgUUUcUU		
CD2 #1<br><br><br><br><br><br>CD2 #2<br><br><br><br><br>CD2 #3	aUUUacaUggaaagcUcaUcUGUUUUA GUACUCUGGAAACAGAAUCUACUA AAACAAGGCAAAAUGCCGUGUUUA UCUCGUCAACUUGUUGGCGAGAUU UU<br><br>aUgaagagcUcagaaUcaaaaGUUUUAG UACUCUGGAAACAGAAUCUACUAAA ACAAGGCAAAAUGCCGUGUUUAUC UCGUCAACUUGUUGGCGAGAUUUU<br><br>gaagaaacaUUgaaaaUcagaGUUUUAG UACUCUGGAAACAGAAUCUACUAAA ACAAGGCAAAAUGCCGUGUUUAUC UCGUCAACUUGUUGGCGAGAUUUU	SaCas9	SBS Genetech
CD3e #1<br>CD3e #2<br>CD3e #3	GggccaUUgUUcccaaUgcU<br>GcaggcaccUgaggcUggga<br>UUacUUUacUaagaUggcgg	SpCas9	Synthego
CD3e #1<br><br><br><br><br>CD3e #2<br><br><br><br><br>CD3e #3	UggggccaUUgUUcccaaUgcGUUUUAG UACUCUGGAAACAGAAUCUACUAAA ACAAGGCAAAAUGCCGUGUUUAUC UCGUCAACUUGUUGGCGAGAUUUU<br><br>aaaaaggagggcUUUaUaagaGUUUUAG UACUCUGGAAACAGAAUCUACUAAA ACAAGGCAAAAUGCCGUGUUUAUC UCGUCAACUUGUUGGCGAGAUUUU<br><br>gcUgggaggggaggagggUUUGUUUUAG UACUCUGGAAACAGAAUCUACUAAA ACAAGGCAAAAUGCCGUGUUUAUC UCGUCAACUUGUUGGCGAGAUUUU	SaCas9	SBS Genetech
TRAC #1	gagaatcaaaatcggtgaat	SpCas9	Synthego
TRAC #1**	GAGTCTCTCAGCTGGTACACGGC	AsCas12a	IDT

**All Sp sgRNAs have 2’-O-methyl on the three terminal nucleotides at both ends and 3’ phosporothioate at the first three bases and last 2 bases. Sa sgRNAs have 2’-O-methyl and 3’ phosporothioate on the three terminal nucleotides at both ends.*

*** crRNA for AsCas12a*

Table S4

List of antibodies, live/dead markers, and compensation beads used for flow cytometry.

Laser	Antigen	Flourophore	Clone	Vendor	Cat#	Volume
**CytoFLEX S V4-B2-Y4-R3 instrument (Beckman Coulter)**						
405nm	CD123	BV421	6H6	Biolegend	306018	Recommended
	Zombie Yellow fixable viability dye	N/A	N/A	Biolegend	423103	Recommended
	Zombie Violet fixable viability dye	N/A	N/A	Biolegend	423113	Recommende d
488nm	CD3e	FITC	OKT3	TonBo Biosciences	35-0037-T100	Recommended
561nm	CD2	PE	RPA-2.10	TonBo Biosciences	50-0029-T025	Recommended
	CD5	PE	UCHT 2	eBioscience	12-0059-42	Recommended
	TCR α/β	PE	IP26	eBioscience	12-9986-42	Recommended
638nm	CD5	APC	UCHT 3	TonBo Biosciences	20-0059-T100	Recommended
	CD271	APC	ME20 .4	Biolegend	345108	Recommended
	CD4	PE-Cy7	RPA-T4	TonBo Biosciences	60-0049-T100	Recommended

	CD8a	APC	HIT8a	Biolegend	300912	Recommended
**NovoCyte Quanteon 4025 (Aligent)**						
405nm	Zombie Yellow fixable viability dye	N/A	N/A	Biolegend	423103	Recommended
561nm	CD123	PE	6H6	eBioscience	12-1239-42	Recommended
637nm	CD19	APC	HIB19	BD	302212	Recommended
	CD271	APC	ME20.4	Biolegend	345108	Recommended
**Compensation**						
SpectraComp Compensation Beads				SLiNG SHOT Bio	SSB-05-A	Recommended
UltraComp Compensation Beads				Invitrogen	01-2222-41	Recommended

Table S5

RNA-sequencing data; DEG list, Predicted off-target sites,TSS coordinates, and Output from search within 10kb.

Go to the excel-file *"Supplemental table 5"*.
